# Clinical Efficacy and Safety of Ezetimibe on Major Cardiovascular Endpoints: Systematic Review and Meta-Analysis of Randomized Controlled Trials

**DOI:** 10.1371/journal.pone.0124587

**Published:** 2015-04-27

**Authors:** Alessandro Battaggia, Alberto Donzelli, Maria Font, Davide Molteni, Antonio Galvano

**Affiliations:** 1 Infofarma Unità Locale Socio Sanitaria 20, Verona, Italy; 2 Azienda Sanitaria Locale di Milano, Milan, Italy; 3 University of Milan, Milan, Italy; 4 University of Palermo, Palermo, Italy; Cardiff University, UNITED KINGDOM

## Abstract

**Background:**

Randomized clinical trials (RCTs) about Ezetimibe's efficacy on patient-oriented outcomes have given discordant results. The aim of this study was to determine the net effect of Ezetimibe and of the widely marketed combination, Ezetimibe+simvastatin, on mortality and morbidity outcomes.

**Methods and Findings:**

We searched for RCT on Ezetimibe using MEDLINE, CCTR, EMBASE, ClinicalTrials.gov databases up to December 2013, Merck and Novartis online registers, and personal communications. Two authors independently selected trials fulfilling these criteria: RCTs comparing Ezetimibe±statin or another lipid-lowering drug against placebo, or against the same lipid-lowering drug at the same dosage, with a follow-up at least 24 weeks and one or more of these outcomes: all-cause mortality, cardiovascular (CV) mortality, stroke, myocardial infarction (MI), cancer, serious adverse events (SAEs); we assessed the risk of bias using the Cochrane checklist. We extracted the data for major clinical events as a dichotomous measure, with the patient the unit of analysis. Pooled analysis was done with random and fixed effect based models. Trials comparing Ezetimibe plus a lipid-lowering drug against the same lipidlowering drug representing the net effect of Ezetimibe, showed a nonsignificant tendency toward damage for cancer, MI, stroke and SAEs. Ezetimibe+simvastatin vs. simvastatin alone showed a stronger tendency towards a higher risk for all-cause death (2.52; 0.65-9.74), CV death (3.04; 0.48-19.21), non-CV death (3.03; 0.12-73.50), MI (1.91; 0.42-8.70), stroke (2.38; 0.46-12.35), cancer (RR 11.11; 0.62-198.29), and SAEs (1.45; 0.95-2.23). Limitations include small numbers of events and inadequate power of the pooling. Trials comparing Ezetimibe+simvastatin vs placebo showed non-significant effects: MI (0.81; 0.66-1.00 p = 0.051), all-cause death (1.02; 0.95-1.09), CV death (0.91; 0.80-1.04), non-CV death (108; 0.99-1.18), stroke (0.86; 0.72-1.04), cancer (1.18; 0.80-1.74), SAEs (1.01; 0.96-1.06).

**Conclusions:**

Ezetimibe±simvastatin had inconsistent effects on important outcomes. No firm conclusions are possible, but findings indicative of damage suggest much more selective use of Ezetimibe±simvastatin.

## Introduction

### Rationale

Ezetimibe (E) is a lipid-lowering agent that inhibits intestinal absorption of dietary cholesterol. The standard dose of 10 mg/day lowers low-density lipoprotein cholesterol (LDL-C) by 15–20% when used alone, in addition to the reduction achieved with statins. There is strong pressure for the continued use of E for the prevention of cardiovascular (CV) disease. However, clinical trials have given discordant results.

The ENHANCE trial [1] found that adding E to simvastatin 80 mg daily in patients with heterozygous familial hypercholesterolemia caused an additional 16.5% reduction in LDL-C, without affecting the mean change in carotid intima-media thickness (CIMT) compared with simvastatin monotherapy. The CV events and all-cause mortality were not reduced (respectively 10 and 2 in the E plus simvastatin arm, 7 and 1 with simvastatin alone). A subgroup analysis showed a lack of effect with E, also in the key subgroup of patients with higher baseline LDL-C [2].

The subsequent SEAS trial [3] showed that E plus simvastatin significantly reduced LDL-C in patients with aortic stenosis, compared with placebo, without affecting the composite primary endpoint of aortic-valve and ischemic events. There was a significantly larger number of cancers and cancer deaths in the E arm. All-cause deaths were 105 in the E group, and 100 in the placebo group (HR 1.04; 95% CI 0.79–1.36).

The ARBITER 6-HALTS trial [4] found that extended-release niacin added to a statin reduced LDL-C less than E plus a statin, but niacin gave significant reductions (regression) in mean and maximal CIMT, whereas E did not reduce either, with a significant difference between the E and niacin groups. Changes from baseline CIMT stratified by quartiles of increasing cumulative drug exposure showed that the quartile with the highest exposure to E experienced unexpected CIMT progression [5]. Moreover, the incidence of major adverse CV events was significantly higher in the E group (9, 5%) than in the niacin group (2, 1%) (P = 0.04), and respectively 7 and 1 patients died. E had an apparent beneficial effect in the open-label SANDS trial [6], which compared aggressive LDL-C lowering or standard therapy among American Indians with type 2 diabetes mellitus. In the intensive management cohort 31% of the participants required the addition of E to statin therapy to reach the prespecified LDL-C target of ≤70 mg/dL. Patients receiving intensive therapy had significant regression of CIMT compared with those treated with the standard regimen, but the trial was not designed to distinguish which intervention was responsible for this. A secondary analysis [7] found that E plus statin had a beneficial effect on CIMT identical to that of statin alone for a similar change in LDL-C. Although SANDS was not powered to address clinical outcomes, the intensively managed patients did not have any reduction in CV events after three years of follow-up compared to individuals receiving standard treatment (5.8% of CV events in the E plus statin group, 3.5% in the statin alone standard group, 3.3% in the statin alone intensive group).

The UK HARP II trial [8] was conducted in preparation for a larger trial (SHARP) to assess the biochemical efficacy, safety, and tolerability of adding E to simvastatin 20 mg/day for patients with chronic kidney disease (CKD). Again, compared with simvastatin 20 mg alone, E plus simvastatin did not offer any advantage, and in fact there were more unfavorable events in this group: five fatal or nonfatal CV events, three deaths and four cancer cases, versus three CV events, no deaths and no cancers with simvastatin alone.

The lack of evidence of patient-oriented benefits has led some authors to state: “Until outcome trials provide additional insights into the effects of E on CV events, this drug should only be considered an expensive tool to provide a cosmetic effect on blood examinations” [9].

The large SHARP trial [10] in CKD patients, with a median follow-up of 4.9 years, showed for the first time a beneficial effect on CV events with E plus simvastatin 20 mg daily, producing a 17% reduction in major atherosclerotic events compared to placebo (11.3% vs. 13.4%), mostly due to a reduction in revascularizations. However, all-cause mortality was not reduced in the E plus simvastatin group (1,142 deaths [24.6%] vs. 1,115 deaths [24.1%] with placebo. RR 1.02; 95% CI 0.94–1.11).

All-cause mortality and total SAEs are considered less subject to bias than some CV outcomes such as revascularizations and even strokes [11–13]. Moreover, the CV benefits reported in SHARP, comparing E plus simvastatin against placebo, might be due exclusively to the effect of the statin. An indirect comparison with the subgroup of pre-dialysis patients in the HPS trial, which used only simvastatin, suggests that this drug alone would have done better. In fact, in HPS the RR of CV events with simvastatin alone was 0.72 (95% CI 0.62–0.84) [14]—namely, there was a significant 28% reduction of CV events—against 17% in SHARP. These results cast doubt on the net benefit of E on CV morbidity and mortality as well as on other safety outcomes [15].

### Objectives

With these considerations in mind, we conducted a systematic review followed by a meta-analysis to assess the benefits and safety of E. To assess its net effect we selected trials in which E alone was compared with placebo and/or in which the combination of E+another lipid-lowering drug was compared with the same lipid-lowering drug at the same dosage, with special focus on E+simvastatin, the most widely marketed combination, versus simvastatin alone. As a complementary analysis we also tested the net effect of E+simvastatin (the only E+statin fixed combination available in Italy) versus placebo.

## Methods

### Protocol and registration

No protocol was published before the review.

### Eligibility

The search included RCTs published up to 31 December 2013. We excluded observational data to minimize the risk of bias. Participants were males or females of all ages and in any clinical situation, in order to detect not only CV efficacy but also important safety endpoints.

Interventions were E alone versus placebo or E plus another drug against the same drug, provided that the same dosage was used in both arms; and, as complementary analysis, E+simvastatin versus placebo.

RCTs were eligible if they reported one or more of the following outcomes: all-cause mortality; CV mortality; stroke; MI; cancer; SAEs (namely any adverse event that results in death, is life-threatening, or requires or prolongs hospital stay, or causes persistent or significant disability/incapacity; any probably related congenital anomaly/birth defect or any other condition which investigators judge to represent significant hazards http://www.hhs.gov/ohrp/policy/advevntguid.html).

We did not include composite CV end-points, because their definitions can vary widely between trials. Trials were eligible if their follow-up was 24 weeks or more; we consider this the shortest time required to detect any major clinical endpoint.

### Information sources

We searched records of published trials in three electronic bibliographic databases: MEDLINE (through PubMed); Central Controlled Trials Register of the Cochrane Collaboration; EMBASE. We also searched for unpublished data in the ClinicalTrials.gov site (www.clinicaltrials.gov), in the online registers of trials compiled by Merck (www.merck.com/mrl/clinical_trials/results.org) and Novartis (www.novartisclinicaltrials.com) and through personal communication with the authors of the trials to retrieve unpublished data. Other sources of published data were the reference lists of selected RCTs and of all the meta-analyses about E published up to 31 December 2013.

### Search

The electronic search was sensitivity-tailored (see S1 Appendix for details of the strategy used). We applied no language restriction.

### Study selection

Two authors (AB and DM) independently selected the trials fulfilling the inclusion criteria and a third (AD) resolved any disagreement. After removing duplicates we then recovered the full text of all selected articles for the final check. We also searched unpublished trials obtained through personal communications.

### Data collection

Two authors (AB and DM) independently extracted the data from each single trial using a standardized item-list and a third author (AD) resolved any disagreement. They independently assessed the quality of the trials using a check-list (see below).

### Data items

We extracted the outcome data for major clinical events (deaths, CV deaths, MI, stroke, SAEs, cancer) as dichotomous measures using the patient as the unit of analysis. We did not consider surrogate end-points as LDL-C variations or the mean change in CIMT because not pertinent to our objectives. The outcome definitions used were those reported for each trial. We calculated the numbers of not-CV deaths by subtracting the number of CV deaths from that of all-cause deaths reported for the individual trials.

### Risk of bias in individual trials

To assess the quality of each trial we used the method described by the Cochrane Collaboration, which considers three levels of risk of bias (high, low, unclear) for any RCT and for some quality items [16] (for details see Figure A in S2 Appendix). We examined outcomespecific items concerning three clinically important safety endpoints (all-cause deaths, SAEs, cancer).

We applied this critical appraisal in subgroup analyses of the pooled data.

### Summary measures

The results are expressed as pooled risk ratios (RR) and the precision as 95% confidence intervals (CI).

### Synthesis of results

We tested heterogeneity within pools using the I^2^ (I squared) statistic [16, 17] in a fixed/inverted variance-based model.

A I^2^ value lower than 25% was considered suggestive of low heterogeneity; between 25–75% was considered medium; more than 75% was considered high. For pooled analysis we used the fixed effect-based Mantel-Haenszel model and also the random effect-based model of Der Simonian and Laird [16].

### Risk of bias across studies

We checked for publication bias using the Peters formal statistical test [18]. This test preserves the statistical power of the more widely used Egger test [19], but with less probability of type I error (false positives). A p value <0.10 was considered as suggesting publication bias (for details see Table A in S3 Appendix).

### Additional analyses

#### Subgroup analyses

in the main analysis we tested for effect modifier variables (subgroup effect) using a meta-regression univariate model [16] with the suspected modifier stratified as dummy variable; a cutoff of 0.05 defined the statistical significance for the coefficient test.

The variable suspected as being an effect modifier in the E+another drug versus the same drug analyses was the comparator/co-treatment. In subgroup analyses we also tested the influence of *risk of bias* on overall results of poolings.

#### Power analysis

We calculated the statistical power of the meta-analysis for SAEs in scenarios of added sample size using the method described by Crowther et al., which assumes effect sizes of future trials consistent with those observed previously—as represented by the current meta-analysis [20] (for details see Table A in S4 Appendix).

#### Missing data analysis

For absolute risk calculations, we used the number of randomized patients as denominator and the number of events reported as numerator. This is one of the most commonly used strategies [21] and assumed in this particular instance that none of the missing participants experienced the event. Like all methods used where there are missing data—this strategy can be biased if the number of missing data is not small and the prognosis of patients not included in analyses differs between arms.

For these reasons we analyzed the number of missing data in trials included in the meta-analysis and, when reported, the reasons why the data were missing. Finally, we conducted a sensitivity analysis to verify the robustness of conclusions in alternative scenarios of missing data (for details see Tables B-H in S5 Appendix).

All analyses were done using the Stata12-SE statistical package.

## Results

### Study selection

We found 331 records (S1 PRISMA Checklist).

After excluding 86 duplicates and 238 trials not meeting the inclusion criteria, 7 RCTs were selected [1, 8, 29, 30, 31, 32, 33] in which the combination of E+another lipid-lowering drug was tested against the same lipid-lowering drug at the same dosage; these were included in the quantitative synthesis (metaanalysis).

We also selected two RCTs [3, 10] which tested E+simvastatin against placebo.

The trials included (with the reasons for exclusion) are listed in Tables A, B and C in S6 Appendix.

We contacted all the authors of the articles included to ask whether they had any unpublished data, but only four of the 18 replied. The responders (see Acknowledgments for the list) all stated they had no unpublished data in their datasets.

### Study characteristics

Table 1 summarizes the baseline characteristics of patients in the seven RCTs E+statin vs. active comparator (main analysis) and in the two RCTs E+simvastatin vs. placebo (complementary analysis). The main analysis enrolled 2,212 people; mean age ranged from 46 to 71 years and males ranged from 41% to 93%. All these trials were small (median sample size 224, min. 50 max.720). The complementary analysis enrolled 11,143 patients (mean ages in SHARP and SEAS trials respectively 62 and 67.5 years, males 62.6 and 61.4 percent).

**Table 1:** Main baseline data of patients in the trials included

Trial (see references in S6 Appendix)	year	age	No. in ezetimibe arm	No. in comparator arm	Ezeti-mibemg/day	Cotreatment (ezetimibe arm)	Cotreatmentmg/day	Comparator (control arm)	Comparatormg/ day	Males(%)*	Diabetes (%)^1^	Coronary artery disease (CHD %)^1^	Peripheral artery disease (PAD%)^1^
Arimura^2^	2012	69.0	25	25	10	atorvastatin	10	atorvastatin	10	70.0%	28.0%	100.0%	
Kouvelos^2^	2012	71.0	126	136	10	rosuvastatin	10	rosuvastatin	10	89.7%	30.2%	49.2%	100.0%
ENHANCE^2^	2008	45.7	357	363	10	simvastatin	80	simvastatin	80	51.4%	1.8%	5.6%	na
UK-HARP-II^2^	2006	60.0	101	102	10	simvastatin	20	simvastatin	20	69.5%	10.8%	na	na
Ballantyne^2^	2004	58.1	201	45	10	atorvastatin	10	atorvastatin	10	41.1%	6.1%	11.8%	3.3%
West^2^	2011	63.5	48	20	10	simvastatin	40	simvastatin	40	55.9%	32.4%	61.8%	100.0%
McKenney^2^	2006	53.5	340	236	10	phenofibrate	160	phenofibrate	160	57.5%	16.1%	na	na
SHARP^3^	2011	62	4650	4620	10	simvastatin	20	placebo	-	62.6%	22.6	2.9%	6.0%
SEAS^3^	2008	67.5	944	929	10	simvastatin	40	placebo	-	61.4%	na	na	na

^1^ percentage of the whole sample

^2^ main analysis

^3^ complementary analysis

na = not available

### Risk of bias within trials

The quality of the seven trials in our main analysis, (Figure A in S2 Appendix), was generally hard to establish because of poor reporting. The random numbers generation was correctly described in 71.4% of trials and the concealment of allocation was satisfactorily reported only in 42.8%. A potential lack of blinding probably did not influence the interception of the outcome death (100% free of bias), but this was not necessarily true for other outcomes, such as CV events (revascularizations, MI, and stroke), cancer and SAEs [11–13]. No trial was reliably free of bias for “blindness” and for the outcome cancer, and only 14.2% were free of bias for SAEs.

The accuracy of the data reported varied from 28.5% to 42.8% for the outcomes considered in the quality analysis. For 71.4% of the trials some problems in selective outcome reporting cannot be excluded, and only in 14.2% can conflict of interest be reasonably excluded. For six trials out of seven (85.7%) the funding source was in fact not clear, and only one (14.2%) was not sponsored by private sources.

The quality of the two trials with placebo as comparator is reported in S2 Appendix. Both were funded by the manufacturer of E; nevertheless SHARP authors stress that the sponsor did not have any influence on trial conduction and data analysis. For the SHARP trial we cannot exclude incomplete data reporting for all three endpoints considered and some pitfalls in the blinding for the outcome cancer; we also noted selective outcome reporting.

For the SEAS trial we cannot exclude inadequate random sequence generation, allocation concealment and blinding in two out of three outcomes considered in our analysis.

### Results of individual trials

Tables A-P in S7 Appendix illustrate the results of individual studies.

### Summary of results

The results of the main analysis (E+drug vs. same drug) and of supplementary analysis respectively (E+simvastatin vs placebo) are expressed using fixed- and random-effect methods and stratified by the comparator, as shown in upcoming figures. Heterogeneity in every pool was substantially low, except for a medium degree for the outcome cancer, so for the majority of the analyses a fixed-effect model (fem) should be adopted (the random effect model—REM—is preferable when there are high levels of unclear heterogeneity within pools [16]: it offers larger confidence intervals, and is therefore a more ‘conservative’ technique).

For “All comparisons” E+drug vs. same drug there was a trend with E towards an increased risk of cancer: RR 3.12 (0.62–15.61) for the fem and RR 2.14 (0.07–64.24) for the rem (this latter model is preferable here considering the moderate degree of heterogeneity (60.1%)).

For all-cause death and not-CV death the effect of E appeared neutral at most: RR 1.03 (0.43–2.44) and RR 1.04 (0.15–7.48) respectively. For CV death, there was a tendency towards a small benefit: RR 0.90 (0.31–2.59). A trend toward damage was observed for MI, stroke and SAEs, with RR 1.37 (0.37–5.01), RR 1.45 (0.43–4.87) and RR 1.24 (0.88–1.73) respectively.

**Fig 1:**
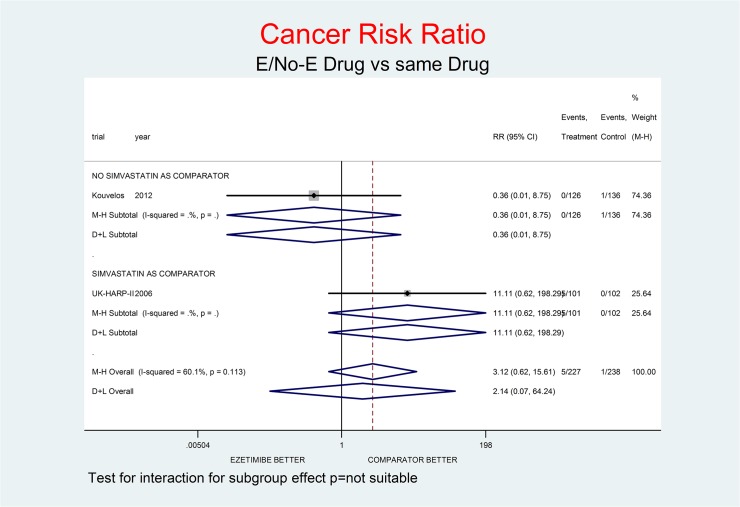
Main analysis: net effect of Ezetimibe on Cancer stratified by comparator (E+lipid-lowering drug against the same lipid-lowering drug at the same dosage; E+simvastatin against simvastatin at the same dosage). The medium degree of heterogeneity (I^2^ = 60.1%) should encourage the use of the random effect method of pooling. No test for interaction was done (only two trials). The overall result, although not significant, shows a definite trend toward damage (212% increment of Cancer Risk, p = 0.167), due to an impressive trend toward damage in the UK-HARP-II trial, where simvastatin was the Not-E drug.

**Fig 2:**
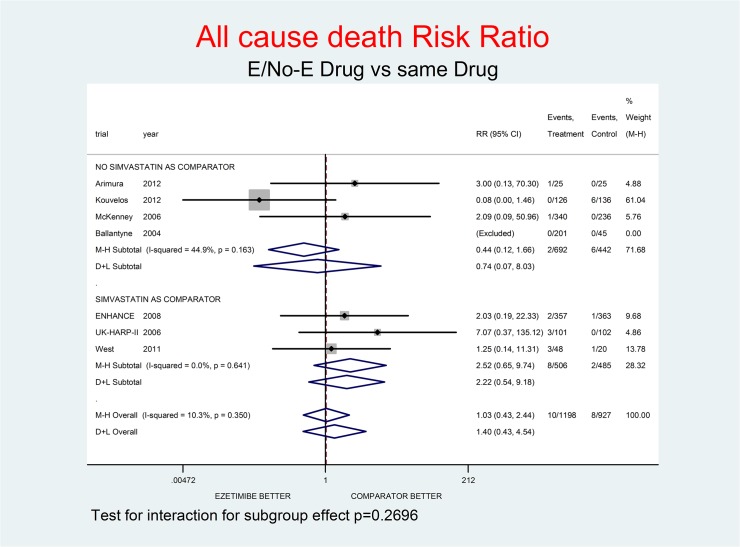
Main analysis: net effect of Ezetimibe on all-cause death stratified by comparator (E+lipid-lowering drug against the same lipid-lowering drug at the same dosage; E+simvastatin against simvastatin at the same dosage). The low degree of heterogeneity (I^2^ = 10.3%) should encourage use of the fixed effect method of pooling. The test for interaction does not show the formal presence of a ‘subgroup effect’ (p = 0.2696); however, the direction of the result is toward damage only in the stratum where simvastatin was the no-E drug, showing a clinically important increment of death risk. The non-significant overall result shows a small trend toward damage (3% increment of death risk, p = 0.947).

**Fig 3:**
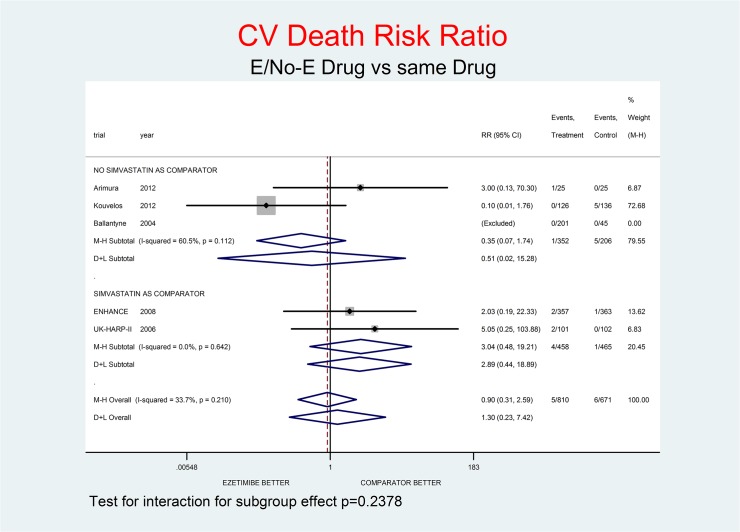
Main analysis: net effect of Ezetimibe on CV death stratified by comparator (E+lipid- lowering drug against the same lipid-lowering drug at the same dosage; E+simvastatin against simvastatin at the same dosage). The low degree of heterogeneity (I^2^ = 33.7%) should encourage the use of the fixed effect method of pooling. The test for interaction does not show the formal presence of a ‘subgroup effect’ (p = 0.2378); however, the direction of the result is toward damage only in the stratum where simvastatin was the no-E drug, showing a clinically important increment of CV death risk. The non-significant overall result shows a trend toward benefit (10% decrement of CV death risk, p = 0.844).

**Fig 4:**
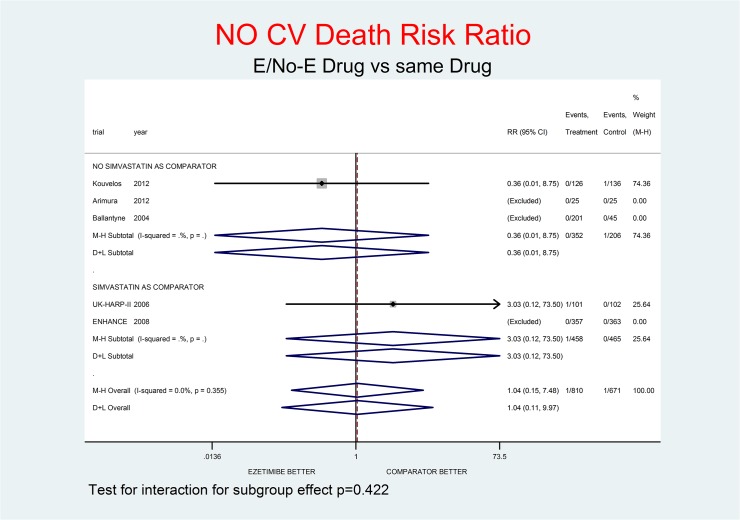
Main analysis: net effect of Ezetimibe on not-CV death stratified by comparator (E+lipid- lowering drug against the same lipid-lowering drug at the same dosage; E+simvastatin against simvastatin at the same dosage). The low degree of heterogeneity (I^2^ = 0.0%) should encourage the use of the fixed effect method of pooling. The test for interaction does not show the formal presence of a ‘subgroup effect’ (p = 0.422); however, the direction of the result is toward damage only in the stratum where simvastatin was the no-E drug, showing a clinically important increment of not-CV death risk. The non-significant overall result shows a small trend toward damage (4% increment of not-CV death risk, p = 0.966). We calculated the number of not-CV deaths by subtracting the number of CV deaths from the number of all-cause deaths reported by each single trial.

**Fig 5:**
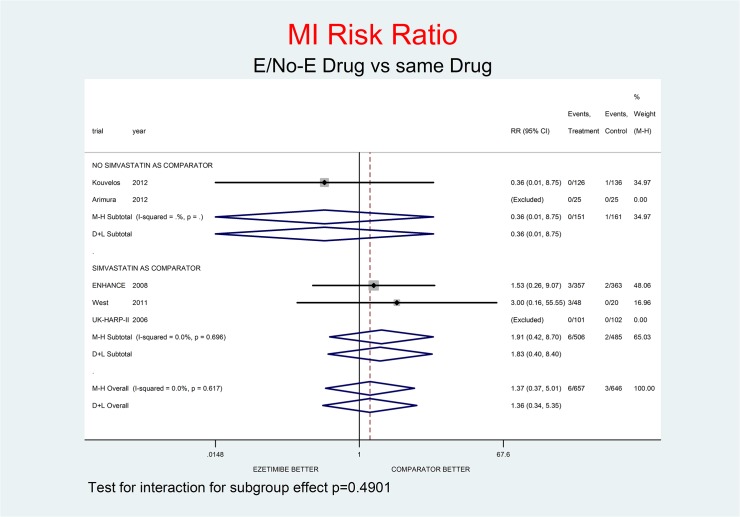
Main analysis: net effect of Ezetimibe on MI stratified by comparator (E+lipid lowering drug against the same lipid-lowering drug at the same dosage; E+simvastatin against simvastatin at the same dosage). The low degree of heterogeneity (I^2^ = 0.0%) should encourage the use of the fixed effect method of pooling. The test for interaction does not show the formal presence of a ‘subgroup effect’ (p = 0.4901); however, the direction of the result is toward damage only in the stratum where simvastatin was the no-E drug, showing a clinically important increment of MI risk. The non-significant overall result shows a trend toward damage (37% increment of MI risk, p = 0.636).

**Fig 6:**
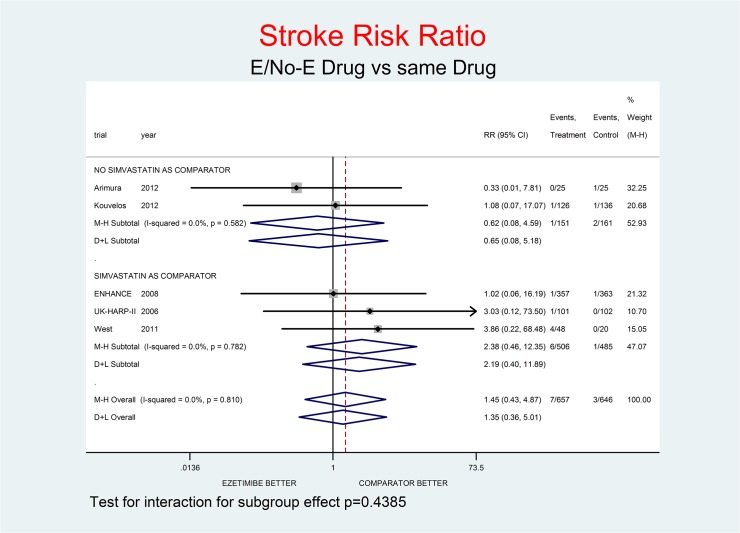
Main analysis: net effect of Ezetimibe on stroke stratified by comparator (E+lipid- lowering drug against the same lipid-lowering drug at the same dosage; E+simvastatin against simvastatin at the same dosage). The low degree of heterogeneity (I^2^ = 0.0%) should encourage the use of the fixed effect method of pooling. The test for interaction does not show the formal presence of a ‘subgroup effect’ (p = 0.4385); however, the direction of the result is toward damage only in the stratum where simvastatin was the no-E drug, showing a clinically important increment of stroke risk. The non-significant overall result shows a trend toward damage (45% increment of stroke risk, p = 0.546).

**Fig 7:**
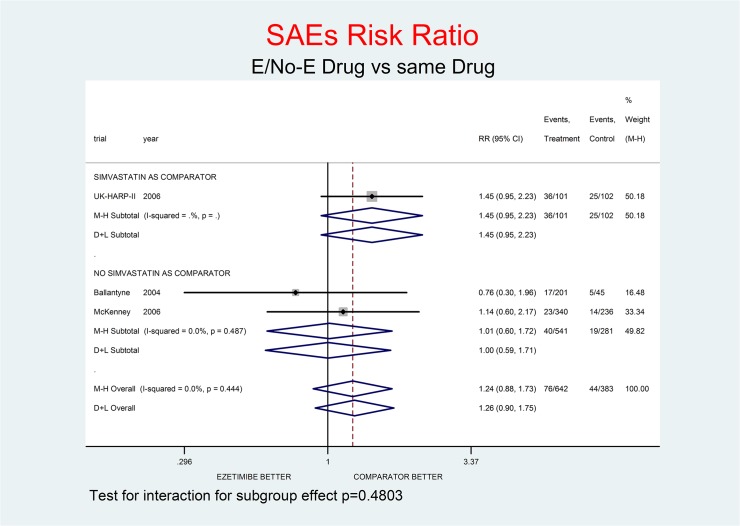
Main analysis: net effect of Ezetimibe on SAEs stratified by comparator (E+lipid- lowering drug against the same lipid-lowering drug at the same dosage; E+simvastatin against simvastatin at the same dosage). The low degree of heterogeneity (I^2^ = 0.0%) should encourage the use of the fixed effect method of pooling. The test for interaction does not show the formal presence of a ‘subgroup effect’ (p = 0.4803); however, the direction of the result is toward damage only in the stratum where simvastatin was the no-E drug, showing a clinically important 45% increment of SAEs risk. The non-significant overall result shows a trend toward damage (24% increment of SAEs risk, p = 0.216). The larger risk of major clinical endpoints was in fact noted in the E arms of trials where E plus simvastatin were compared with simvastatin alone (Figs 1–7).

Tests for interaction (Figs 1–7) do not show formal “subgroup effects” when stratifying by “simvastatin used as comparator” (all p values were >0.05); nevertheless there was a constant trend toward damage in all meta-analyses of the strata with simvastatin as comparator.

Figs 1–7 represent meta-analyses of the trial results in main analysis (E/No-E drug versus same drug). The forest plots illustrate the pooling results using both fixed and random models. Tests for interaction do not show formal “subgroup effects” stratifying by “simvastatin use as comparator” (all p values were >0.05); nevertheless there was a constant trend toward damage in all meta-analyses of the strata characterized by simvastatin use as “No-E Drug”.

E10+simvastatin represents the only fixed-dose combination currently available on the Italian market. This combination showed a strong tendency towards a higher risk for all the outcomes (Figs 1–7). The increase in the risk of cancer seems large (RR 11.11; 0.62–198.29), but it came from only one trial, with small numbers of events. The E+simvastatin combination also increased the risk for all outcomes (all-cause death RR 2.52, 95% CI 0.65–9.74; CV deaths 3.04, 0.48–19.21; not-CV deaths 3.03, 0.12–73.5; MI 1.91, 0.42–8.70; stroke 2.38, 0.46–12.35; SAEs 1.45, 0.95–2.23).

**Fig 8:**
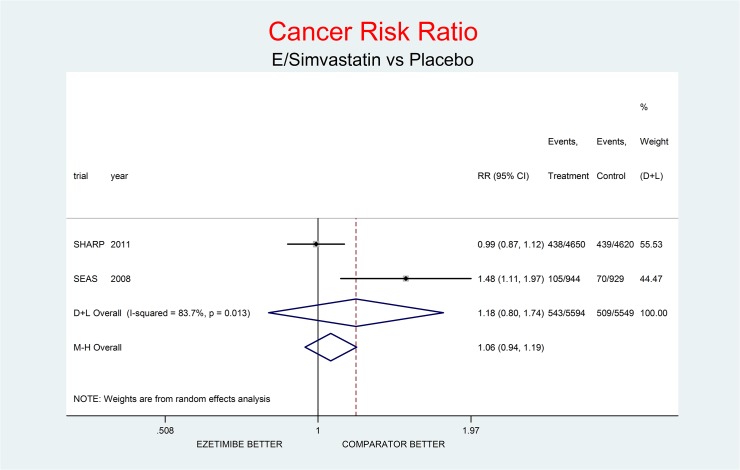
Complementary analysis: net effect of the Ezetimibe/Simvastatin combination (E+Simvastatin against placebo) on cancer. The simvastatin dosage was different in the two trials (20 mg/day in SHARP, 40 mg/day in SEAS). The high degree of heterogeneity (I^2^ = 83.7%) should encourage use of the random effect method of pooling. The non-significant overall result shows a trend toward damage (18% increment of cancer risk, p = 0.395).

**Fig 9:**
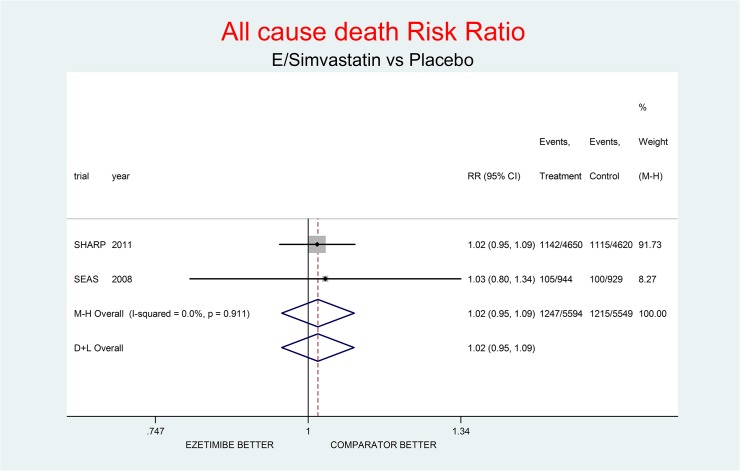
Complementary analysis: net effect of the Ezetimibe/Simvastatin combination (E+simvastatin against placebo) on all-cause death. The simvastatin dosage was different in the two trials (20 mg/day in SHARP, 40 mg/day in SEAS). The low degree of heterogeneity (I^2^ = 0.0%) should encourage the use of the fixed effect method of pooling. The nonsignificant overall result shows a small trend toward damage (2% increment of all-cause death risk, p = 0.596).

**Fig 10:**
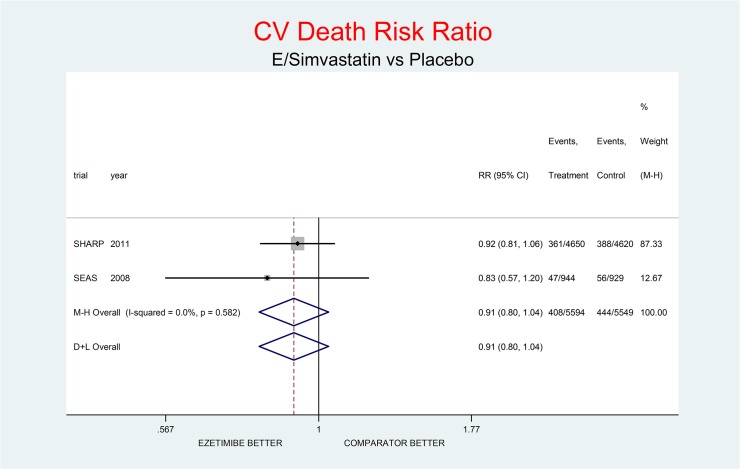
Complementary analysis: net effect of the Ezetimibe/Simvastatin combination (E+Simvastatin against placebo) on CV death. The simvastatin dosage was different in the two trials (20 mg/day in SHARP, 40 mg/day in SEAS). The low degree of heterogeneity (I^2^ = 0.0%) should encourage the use of the fixed effect method of pooling. The nonsignificant overall result shows a trend toward benefit (9% decrement of CV death risk, p = 0.162).

**Fig 11:**
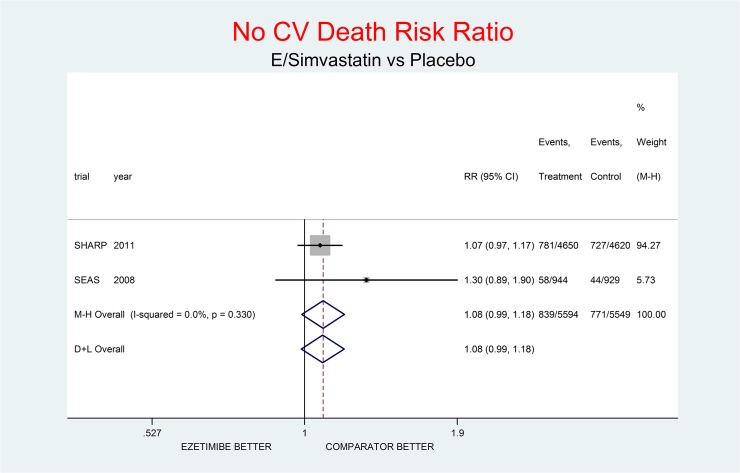
Complementary analysis: net effect of the Ezetimibe/simvastatin combination (E+simvastatin against placebo) on not-CV death. The simvastatin dosage was different in the two trials (20 mg/day in SHARP, 40 mg/day in SEAS). The low degree of heterogeneity (I^2^ = 0.0%) should encourage the use of the fixed effect method of pooling. The nonsignificant overall result shows a trend toward damage (8% increment of not-CV death risk, p = 0.091). We calculated the number of not-CV deaths by subtracting the number of CV deaths from the number of all-cause deaths reported by each single trial.

**Fig 12:**
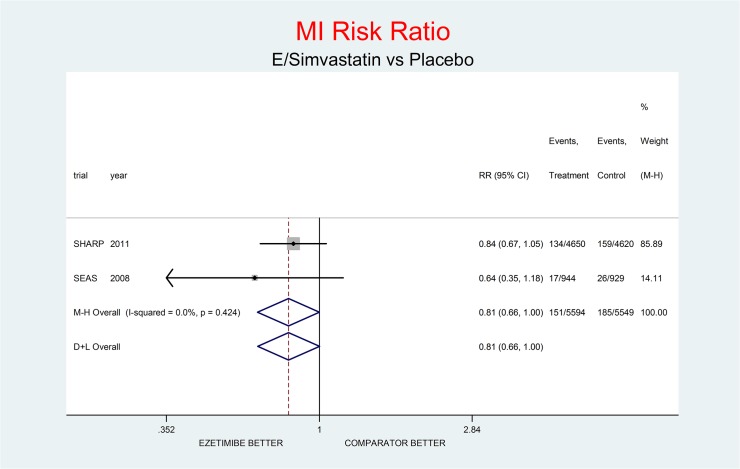
Complementary analysis: net effect of the Ezetimibe/simvastatin combination (E+simvastatin against placebo) on MI. The simvastatin dosage was different in the two trials (20 mg/day in SHARP, 40 mg/day in SEAS). The low degree of heterogeneity (I^2^ = 0.0%) should encourage the use of the fixed effect method of pooling. The non-significant overall result shows a trend toward benefit (19% decrement of MI risk, p = 0.051).

**Fig 13:**
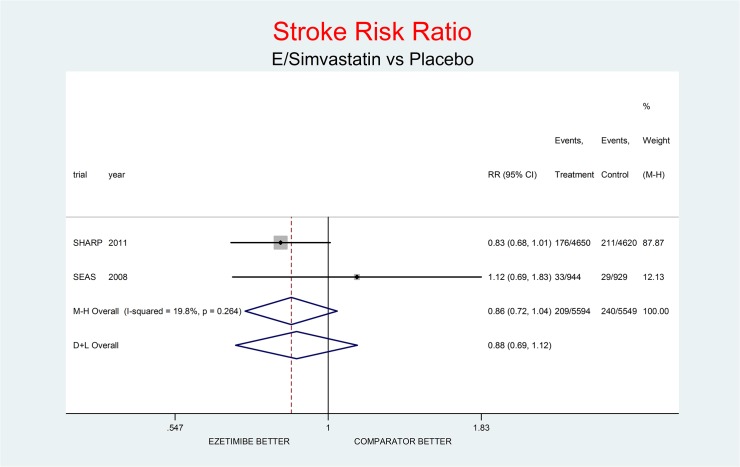
Complementary analysis: net effect of the Ezetimibe/simvastatin combination (E+simvastatin against placebo) on Stroke. The simvastatin dosage was different in the two trials (20 mg/day in SHARP, 40 mg/day in SEAS). The low degree of heterogeneity (I^2^ = 19.8%) should encourage the use of the fixed effect method of pooling. The non-significant overall result shows a trend toward benefit (14% decrement of Stroke risk, p = 0.115).

**Fig 14:**
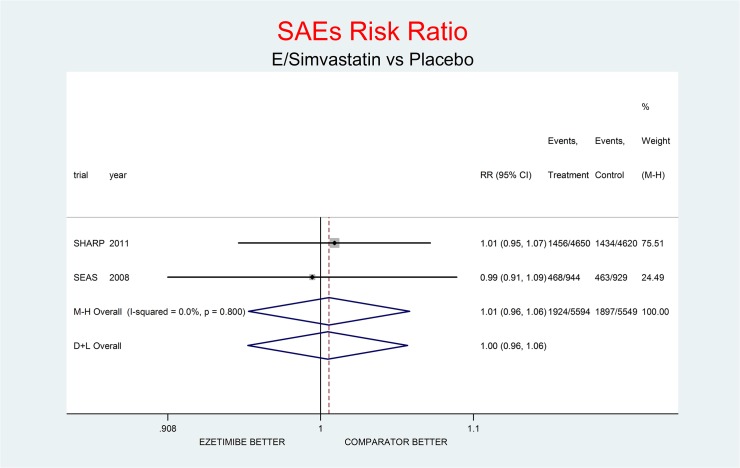
Complementary analysis: net effect of the Ezetimibe/simvastatin combination (E+simvastatin against placebo) on SAEs. The simvastatin dosage was different in the two trials (20 mg/day in SHARP, 40 mg/day in SEAS). The low degree of heterogeneity (I^2^ = 0.0%) should encourage the use of the fixed effect method of pooling. The non-significant overall result shows a small trend toward benefit (1% increment of SAEs risk, p = 0.837).

As complementary analysis we meta-analyzed the results of the SHARP [10] and SEAS [3] trials (Figs 8–14) bearing in mind the limits of the comparison due to differences in the recruited high CV risk populations and the different dosages of the coadministrated simvastatin.

Figs 8–14 represent meta-analyses of the trial results in the complementary analysis (E/Simvastatin vs. placebo). Forest plots illustrate the pooling results of the two trials using both fixed and random models.

In SHARP and SEAS E+simvastatin did not reduce all-cause death (RR respectively 1.02, 95% CI 0.94–1.11; and 1.04, 0.79–1.36), though at the same time there was a tendency towards reduction of the risk of CV death (RR 0.93, 95% CI 0.80–1.07; and 0.83, 0.56–1.22 respectively) and towards an increase of the risk on non-CV death (RR 1.09, 95% CI) 0.98–1.21; and 1.26, 0.85–1.86 respectively).

The meta-analysis of the two trials showed a null effect of E+simvastatin for allcause death (RR 1.02, 95% CI 0.95–1.09) and SAEs (RR 1.01, 95% CI 0.96–1.06); a trend toward advantage for MI (RR 0.81, 95% CI 0.66–1.00), stroke (RR 0.86, 95% CI 0.72–1.00) and CV deaths (RR o.91, 95% CI 0.80-1-04) and a trend toward damage for non-CV death (RR 1.08, 95% CI 0.99–1.18) and for cancer (RR 1.18, 95% CI 0.80–1.74).

Table A in S2 Appendix illustrates a subgroup analysis stratified by three tertiles of quality of reports. We used the distribution of the sum of “high risk of bias” and “doubtful risk of bias” judgments for any trial. The trials were therefore stratified as “generally low risk of bias” (first and second tertile) and “generally high risk of bias” (third tertile).

### Risk of bias across trials

Table A in S3 Appendix illustrates for main analysis the “small trials effect” analysis with the Peters test [18]. The p value indicated no “small trials effect”. This may suggest absence of publication bias; but all formal tests for publication bias can give false-negative results when there is only a small number of comparisons, as in this case [16].

### Additional analysis

Table A in S4 Appendix illustrates the point results of the comparisons E plus simvastatin vs. simvastatin for SAEs: the current sample size and the one that needs to be added to the meta-analysis to reach statistical significance for the same results at power ≥80% and a usual level of confidence (95%).

Tables B-H in S5 Appendix illustrate for main analysis the analysis of missing data (61 patients, corresponding to a 2.75% prevalence of missing data in our pool (61/2212. View Appendix 5 for methodological details).

## Discussion

### Summary of evidence

This systematic review found only seven RCTs [1, 8, 22, 23, 24, 25, 26] about E+ a lipid-lowering drug against the same lipid-lowering drug with follow-up long enough to reasonably intercept major clinical CV endpoints (≥24 weeks), in addition to two large RCTs against placebo [3, 10].

Data about E’s efficacy on CV outcomes are scarce because the majority of small trials focused on the safety profile and/or the lipid-lowering efficacy of E, with a short follow-up.

After having explored a wide range of sources of published and unpublished evidence and formally excluded publication bias for the outcomes considered, we can reasonably claim to have found most of the data so far available.

### The net effect of E

E was always used together with other lipid-lowering drugs, mainly statins. In the absence of direct comparisons of E monotherapy against placebo, we assumed as representative of its net effect that produced in trials where the E+lipid-lowering drug combination was compared with the same lipid-lowering drug at the same dosage.

For all comparisons in which E was added to another lipid-lowering drug, the combination showed no overall favorable effect compared to the lipid-lowering drug alone. E had a neutral effect for two major endpoints (all-cause and non- CV death), a not-significant trend toward benefit for CV death and notsignificant trends toward damage for the other endpoints (cancer, MI, stroke, SAEs) (Figs 1–7).

The two big RCTs SHARP [10] and SEAS [3] demonstrate the net effect of the combination of E+simvastatin vs. placebo. In the SHARP trial [10] high CV risk patients with CKD were randomized to E+simvastatin 20 mg daily (4650) or to placebo (4620), with 4.9 years of follow-up. In the SEAS trial [3] 1873 high CV risk patients with mild-to-moderate, asymptomatic aortic stenosis were randomized to E+simvastatin 40 mg daily (944) or to placebo (929), with 4.35 years of follow-up.

The results on composite CV endpoints were contradictory. In SHARP there was a 17% decrease in the composite primary endpoint (any major atherosclerotic event), mainly due to a reduction of revascularization procedures and secondly to a reduction in the risk of non-hemorrhagic stroke. In SEAS E+simvastatin did not reduce the primary endpoint of combined aortic valve events and ischemic events in patients with aortic stenosis.

The meta-analysis of SHARP and SEAS results (Figs 8–14). confirms the doubts about the efficacy/safety profile of E+simvastatin, showing at best a null effect of E+simvastatin for all cause death and SAEs, a trend toward advantage for MI, stroke and CV deaths, and a trend toward damage for non-CV death and for cancer (see above). None of these comparisons were significant at the usual level of confidence (95%).

Other trials which recruited only CKD patients allow an indirect comparison with SHARP outcomes. Unfortunately only two small trials [27, 28] tested outcomes examined in our meta-analysis, as the CKF subgroup [29] in the HPS trial was only evaluated for major CV composite events. In [27] simvastatin10-40 mg + diet was compared with diet alone in 17 pre-dialytic patients, and in the PERFECT trial [28] simvastatin 5–20 mg was compared with placebo in 107 patients on dialysis. Pooling the results for these small trials, for the purpose of discussion—and aware of the limits due to the different doses of the drugs—shows a trend toward a large benefit for all-cause deaths (RR 0.26; 95% CI 0.03–2.27), for CV death (RR 0.26; 95% CI 0.03–2.27) and for non-CV death (RR 0.20; 95% CI 0.02–1.72). So, at least in an indirect comparison with these small trials, CKD patients did not seem to benefit from the addition of E to simvastatin alone.

In our analysis a trend toward damage related to E use appeared clinically relevant for trials where E plus simvastatin was compared with simvastatin alone, the most frequent active comparator, at the same dosage [2, 8, 25] (Figs 1–7). The reasons are not clear, and the confidence intervals do not exclude a chance effect.

The other four trials that compared E+another lipid-lowering drug vs. the same lipid-lowering drug [22, 23, 24, 26] do not permit credible conclusions because of the small number of comparisons, the different drugs combined with E, the heterogeneous results, and the unusual setting and cases [23].

### The power of this search and the implications

Failure to find a significant p-value might mean either that there was no real effect, or that the meta-analysis had inadequate power to detect an effect, as the number of events and trials included was small. When there is a clinically meaningful effect with a statistically non-significant p-value (as in our case), this should cautiously suggest that too few and too small studies are available to answer the question properly. However, it is important to highlight the coherence of some findings more than their statistical significance. The ability of E to lower the surrogate endpoint of LDL-C seems of little value.

Other drugs initially celebrated for their ability to lower LDL-C were dropped because of disappointing clinical results. Examples include torcetrapib plus atorvastatin vs. atorvastatin [30] (CV outcome HR 1.25; 95% CI 1.14–2.19; all-cause death 1.58; 95% CI 1.14–2.19); dalcetrapib plus statin vs. statin [31] (CV outcome HR 1.04; 95% CI 0.93–1.16); niacin plus simvastatin vs. simvastatin [32] (CV outcome HR 1.02; 95% CI 0.87–1.2; all-cause death 1.16; 95% CI 0.87–1.56). It is worth recalling that niacin defeated E in a head-to-head comparison [5], while lowering LDL-C less than E—and there are other examples.

### Potential dangers of E

Some authors have stressed the lack of consistent data about the potential dangers of E, whose effect in the progression of atherosclerosis is very controversial [1, 5, 7, 25]. The final results of the ARBITER 6-HALTS trial indicated that E compared with niacin did not reduce mean CIMT, and in fact greater cumulative E exposure was related to progression of CIMT [5]. In the ENHANCE trial in subjects with familial hypercholesterolemia treated with simvastatin the CIMT progressed in both arms, but more in that with E [1]. In the SANDS trial there were similar reductions in CIMT in the statin and E plus statin arms [7]. In patients with peripheral arterial disease [25] E seemed actually to promote the progression of atherosclerosis.

In the SHARP trial E plus simvastatin compared with placebo significantly reduced the risk of revascularization and of non-hemorrhagic strokes [10].

However, the question remains why the efficacy of E for the surrogate endpoint ‘revascularizations’ was not related to any clinical benefit in MI and coronary mortality in subjects at high baseline risk for these endpoints.

Another important question regards the higher risk of cancer first noted in the SEAS trial [3], further confirmed as a trend in all comparisons in this metaanalysis (Fig 1). The question was discussed in 2008 about the preliminary results of the SHARP and IMPROVE IT trials, excluding any sound evidence that E can increase the risk of cancer. The paper also pooled the results of the SEAS, SHARP and IMPROVE IT data, finding a significantly larger number of deaths from cancer (p = 0.007), disregarded as biased [33], but that might indicate a promoting effect of E on the progression of existing cancers.

Because of E’s controversial risk/benefit profile, some authors argued that its use should be stopped in clinical practice until the research results produce more convincing data [34]. This meta-analysis found a trend towards net damage for E, and no convincing results for E+simvastatin vs. placebo.

At the time of writing, the results of the IMPROVE-IT trial are not yet available. This is a large multicenter double-blind randomized trial, designed to explore the efficacy of E+simvastatin 40 mg vs. simvastatin 40 mg alone in acute coronary syndrome patients [ClinicalTrials.gov Identifier NCT00202878].

IMPROVE IT will be the third big clinical trial (with SEAS and SHARP) adopting non-surrogate endpoints, validated diagnoses and a larger sample than that of the seven trials against active comparator in the present meta-analysis. Therefore it will provide important information about the true effect of E.

Preliminary IMPROVE-IT data, presented on the internet platform via videoconference on November 20, 2014, show a modest benefit of E+simvastatin vs. simvastatin alone on non-fatal CV endpoints, but a null result on all-cause death (15.4% seven-year event rates for E+simvastatin vs. 15.3% with simvastatin alone). If these data are confirmed, it will be another example where the E arm shows more deaths than the simvastatin alone or placebo arms in large or medium-size RCTs [such as 1, 3, 8, 10]. The low odds that this small but repeated increase in deaths happens by chance calls for close evaluation of the total SAEs, not just those that investigators believe to be drug related.

The other reasons for concern include the premature study-drug discontinuation: 42% in both arms. In any case the expected results of IMPROVE-IT do not confirm the excess of cancer risk reported in a previous smaller trial [8].

Until the official figures for the IMPROVE-IT trial are available, this metaanalysis is the first post-hoc evaluation of the effectiveness and safety of E with regard to major clinical endpoints.

### Limitations

#### Publication bias

Our review does not seem to suffer publication bias (see Table A in S3 Appendix), but the techniques employed to ascertain the presence of bias have limited power when there is only a small number of trials. It is not possible therefore to exclude publication bias altogether, given the number of trials—fewer than ten [16]—in this meta-analysis.

#### Misclassification

Only two trials (SHARP and SEAS) of those included in our meta-analysis were tailored on major “centrally detected” CV endpoints (the remaining trials reported those outcomes as “SAEs” or as “drop out due to adverse effects”). It is therefore possible that for some endpoints classified as adverse events the accuracy of diagnosis might have been poor because of the methods used for their interception (e.g. telephone surveys to check for protocol violators or relying on relatives’ answers instead of diagnoses validated by a central blinded committee as in SHARP and in SAES trials). Even though the risk of misclassification is likely bigger for non-fatal endpoints such myocardial infarction, stroke and cancer, the results of this meta-analysis shows for E/simvastatin associations a trend toward harm compared with simvastatin alone, regarding all considered fatal and non fatal endpoints.

## Conclusions

- E has not shown any net advantage in reducing major clinical endpoints, even suggesting a tendency toward damage for some of them. Moreover, when E+simvastatin (the most widely marketed Ecombination) was compared to simvastatin alone, the E arm showed a tendency towards higher risk ratios for every outcome. But the small number of events in these trials does not permit any firm conclusion.
- For drugs licensed to prevent CV events and reduce mortality, like lipidlowering drugs, efficacy should rely on clinically relevant endpoints instead of surrogate ones. More than ten years after E was first marketed, this meta-analysis suggests that reimbursement should only be granted for drugs with proven efficacy on the hardest endpoints.

This means, for E, until the new trial IMPROVE-IT provides clinical data strong enough to reverse the unfavorable trend found in this metaanalysis and to prove a clear overall benefit.

## Supporting Information

S1 PRISMA Checklist PRISMA Checklist. (TIF)

S1 Appendix Search methods. (DOCX)

S2 Appendix Quality assessment of the trials included. (DOCX)

S3 Appendix Publication bias (small trials effect analysis. (DOCX)

S4 Appendix Power analysis for SAEs endpoint in E+simvastatin vs. simvastatin comparisons. (DOCX)

S5 Appendix Management of missing data. (DOCX)

S6 Appendix List of trials included and excluded. (DOCX)

S7 Appendix Individual trial results and meta-analyses. (DOCX)
