# Supplementary figures and images for: Clinical Efficacy and Safety of Ezetimibe on Major Cardiovascular Endpoints: Systematic Review and Meta-Analysis of Randomized Controlled Trials
Source: PLoS One. 2015 Apr 27;10(4):e0124587. doi: 10.1371/journal.pone.0124587 (PMC4411142; doi:10.1371/journal.pone.0124587)

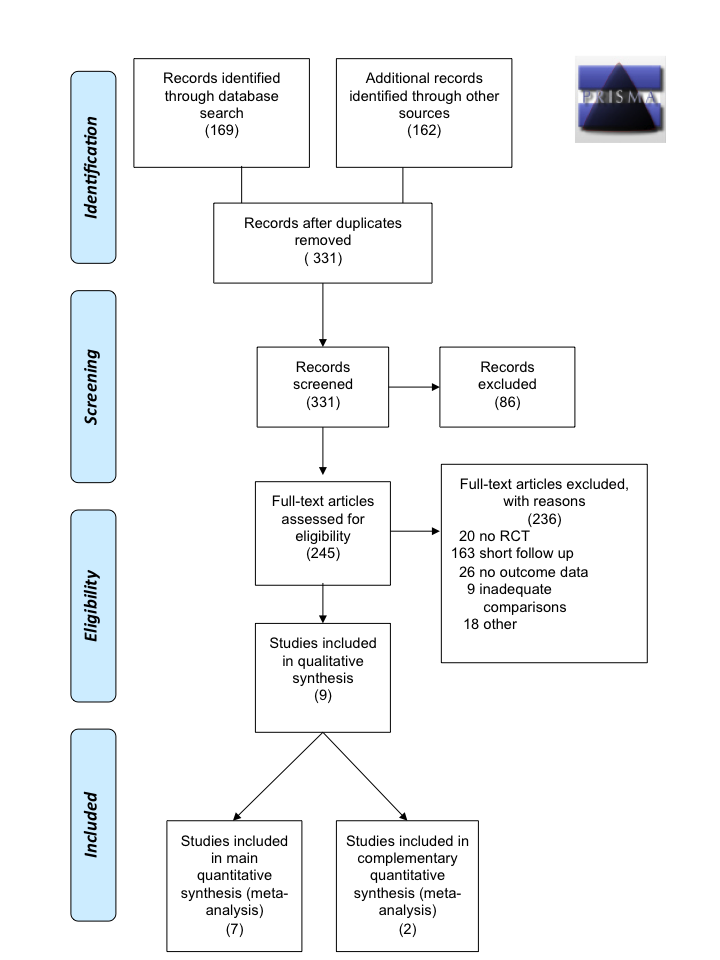

Supplement: S1 PRISMA Checklist — (TIF) [file pone.0124587.s001.tif]
