# Supplementary material for: Clinical Efficacy and Safety of Ezetimibe on Major Cardiovascular Endpoints: Systematic Review and Meta-Analysis of Randomized Controlled Trials
Source: PLoS One. 2015 Apr 27;10(4):e0124587. doi: 10.1371/journal.pone.0124587 (PMC4411142; doi:10.1371/journal.pone.0124587)
Supplement: S1 Appendix — (DOCX) [file pone.0124587.s002.docx]

**S1 Appendix – Search Methods**

**1. ELECTRONIC SEARCH**

1. We used a search strategy for randomized controlled trials (RCT) in Medline through Pubmed. The same search was then modified for RCT searches in other databases (EMBASE, Controlled Cochrane Trial Register - CCTR).

The query was adapted from Robinson & coll. [Robinson KA, Dickersin K. Development of a highly sensitive search strategy for the retrieval of reports of controlled trials using PubMed. International journal of epidemiology 2002;31:150-153].

*((ezetimibe[tiab] AND "Cholesterol, LDL"[Mesh]))*

*AND*

*((Randomized controlled trial[pt] OR controlled clinical trial[pt] OR randomized controlled trials[mh] OR random allocation[mh] OR double blind method[mh] OR single blind method[mh] OR cross-over studies[mh] OR multicenter study[pt]) NOT (animal[mh] NOT human[mh]))*

1. We used the free text strategy

*Ezetimibe*

for RCT on-line searches in Clinical Trials registers ([www.clinicaltrials.gov](http://www.clinicaltrials.gov), [www.merck.com/mrl/clinical_trials/results.org](http://www.merck.com/mrl/clinical_trials/results.org) and [www.novartisclinicaltrials.com](http://www.novartisclinicaltrials.com)

3. We searched meta-analyses for any other published data concerning ezetimibe in the references cited. The strategy in Pubmed for meta-analysis searching was

*Ezetimibe[tiab] AND meta-analysis[pt]*

**2. PERSONAL COMMUNICATIONS**

We contacted the authors of the trials included by mail using a formal request.
