# Supplementary material for: Clinical Efficacy and Safety of Ezetimibe on Major Cardiovascular Endpoints: Systematic Review and Meta-Analysis of Randomized Controlled Trials
Source: PLoS One. 2015 Apr 27;10(4):e0124587. doi: 10.1371/journal.pone.0124587 (PMC4411142; doi:10.1371/journal.pone.0124587)
Supplement: S2 Appendix — (DOCX) [file pone.0124587.s003.docx]

**S2 Appendix - Quality Assessment of the trials included**

1. **TRIAL QUALITY**

Trial Quality was assessed using the tool described in the Cochrane Handbook [reference 16 in main text]

Figure A in S2 Appendix


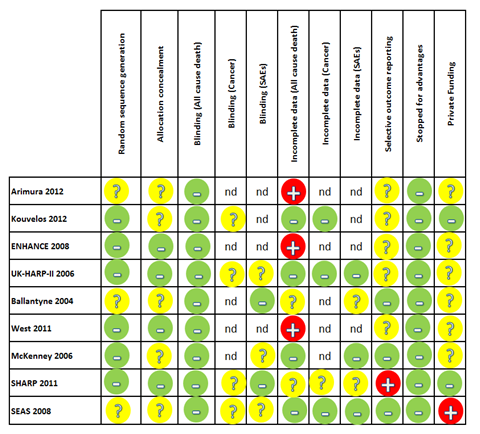


Note: nd indicates that the outcome was not reported; red indicates High risk of Bias, green Lower Risk of bias, and yellow Unclear risk of bias

# Quality of trials included in main analysis

Quality was lower-medium grade in the seven trials in main analysis (Arimura, Kouvelos, ENHANCE, UK-HARP-II, Ballantyne, West, McKenney). The random numbers generation was satisfactorily described for 71.4%, but not for 28.5 %. The concealment of allocation was also inadequately reported (only 42.8% was certainly free from bias). Blinding was very good for the outcome death (100%), but very bad for the outcomes cancer (0%) and SAEs (14.2%). Only for 28.5% of the trials can we not exclude problems in selective outcome reporting and some conflicts of interest can be excluded only in 14.2%.

# Quality of trials included in complementary analysis

In the SHARP trial we cannot exclude incomplete data reporting for all three endpoints and cannot exclude problems of blinding for the outcome cancer; that trial also suffered selective outcome reporting. SEAS was funded by a private sponsor; for this trial we cannot exclude inadequate random sequence generation, inadequate allocation concealment and inadequate blinding for 2/3 of the outcomes considered in our analysis.

1. **META- ANALYSIS STRATIFIED BY RISK OF BIAS**

We verified the influence of poor trial quality on the results of our main analysis [reference 16 in main text] in subgroup analyses through a test for interaction, checking the absence of an “effect modifier” for every endpoint. We used the distribution of the sum of “high risk of bias” and “doubtful risk of bias” judgments for any trial. The trials were therefore stratified as “generally lower risk of bias” (first and second tertile) and “generally high risk of bias” (third tertile).The results are illustrated below. All analyses were negative for subgroup effect (test for interaction p >0.05)

| **Table A in S2 Appendix**  **Meta-analyses stratified by risk of bias**  **Risk Ratio (CI 95%)** | | | | |
| --- | --- | --- | --- | --- |
| Outcome | All groups | Lower -medium risk of bias | High risk of bias | Test for interaction (subgroup effect) p |
| Cancer | RR 3.12  (0.62-15.61) | RR 3.12  (0.62-15.61) | Nd^1^ | Na |
| All-cause death | RR 1.03  (0.43-2.44) | RR 0.93  (0.37-2.30) | RR 3.00  (0.13-70.30) | P=0.599 |
| CV death | RR 0.90  (0.31-2.59) | RR 0.74  (0.23-2.37) | RR 3.00  (0.13-70.30) | P=0.581 |
| Non-CV death | RR 1.04  (0.15-7.48) | RR 1.04  (0.15-7.48) | Nd^2^ | Na |
| Myocardial infarction | RR 1.37  (0.37-5.01) | RR 1.37  (0.37-5.01) | Nd^3^ | Na |
| Stroke | RR 1.45  (0.43-4.87) | RR 1.98  (0.49-8.04) | RR 0.33  (0.01-7.81) | P=0.410 |
| SAEs | RR 1.24  (0.88-1.73) | RR 1.24  (0.88-1.73) | Nd^4^ | Na |

**Nd, not defined**

**Na, not applicable**

**1 For the endpoint cancer no subgroup analysis stratified by quality can be done because both trials were at lower -medium risk of bias**

**2 For Non-CV death no subgroup analysis stratified by quality can be done because the Arimura results cannot be calculated (0/0)**

**3 For the endpoint myocardial infarction no subgroup analysis stratified by quality can be done because the Arimura results cannot be calculated (0/0)**

**4 For the endpoint SAEs no subgroup analysis stratified by quality can be done because all trials were at lower-medium risk of bias.**
