# Supplementary material for: Clinical Efficacy and Safety of Ezetimibe on Major Cardiovascular Endpoints: Systematic Review and Meta-Analysis of Randomized Controlled Trials
Source: PLoS One. 2015 Apr 27;10(4):e0124587. doi: 10.1371/journal.pone.0124587 (PMC4411142; doi:10.1371/journal.pone.0124587)
Supplement: S3 Appendix — (DOCX) [file pone.0124587.s004.docx]

**S3 Appendix - Publication bias (small trials effect analysis)**

**SMALL TRIAS EFFECT ANALYSIS USING PETERS TEST**

Funnel plot analysis is particularly difficult when there are only small numbers of trials - less than ten [reference 16 in main text] - as in our main analysis for all endpoints. So we have not constructed funnel plots and only verified (for our main analysis) the absence of publication bias using the Peters formal test [reference 18 in main text] . This test preserves the statistical power of the more widely used Egger test [reference 19 in main text] .but with less probability of type I error (false positives). Table 1 in Appendix 3 illustrates the results [reference 18 in main text] As with all the statistics currently used for detection of “small trial effects”, we must nevertheless bear in mind that these tools work with lower statistical power, so false negatives are therefore possible, especially when there are only a few trials (as in our case). A p value <0.10 is generally considered to suggest publication bias, so our analysis is not compatible (see Table 1) with the presence of publication bias.

| **Table A in S3 Appendix**  **Peters test for publication bias** [reference 18 in main text] | | | |  |  |
| --- | --- | --- | --- | --- | --- |
| **Outcome** | **No. of**  **trials** | **Tau^2^**  **(odds ratio)** | **df** | **t** | **P** |
| Cancer | 2 | nd | nd | nd | nd |
| All-cause death | 7 | 0.1149 | 5 | 0.578 | 0.5883 |
| CV death | 5 | 0.6709 | 3 | 0.588 | 0.5979 |
| Non-CV death | 5 | 0 | 3 | 0.229 | 0.8336 |
| Myocardial infarction | 5 | 0 | 3 | 0.829 | 0.4679 |
| Stroke | 5 | 0 | 3 | -0.08 | 0.9413 |
| SAEs | 3 | 0 | 1 | 0.445 | 0.7335 |
