# Supplementary material for: Clinical Efficacy and Safety of Ezetimibe on Major Cardiovascular Endpoints: Systematic Review and Meta-Analysis of Randomized Controlled Trials
Source: PLoS One. 2015 Apr 27;10(4):e0124587. doi: 10.1371/journal.pone.0124587 (PMC4411142; doi:10.1371/journal.pone.0124587)
Supplement: S4 Appendix — (DOCX) [file pone.0124587.s005.docx]

**S4 Appendix - Power Analysis for the SAEs endpoint in E/simvastatin vs. simvastatin comparisons**

While there is a general perception that meta-analysis has high power for testing the main effect, this is not altogether correct [reference 17 in main text] . Of course this is particularly important when - as in our case – there is only a small number of pooled trials. The failure to find a statistically significant p-value could of course mean that the effect (if any) is small, but could also mean that the meta-analysis had poor power to detect even a large effect. So, when there is a clinically meaningful effect with a non-significant P value, we must cautiously consider that too few trials have been organized so far to answer the question of interest correctly. This concept must also be applied, of course, to the subsidiaries of a meta-analysis, such as subgroup analyses and meta-regressions.

Finally, it is particularly important for comparisons regarding the strata in which the association E/simvastatin was compared with simvastatin alone, for which our meta-analysis revealed the worst and largest results for all endpoints. For our main analysis we calculated the statistical power of the meta-analysis in scenarios of added sample size using the method described by Crowter & coll, which assumes for future studies effect sizes consistent with those observed previously, as represented by the current meta-analysis [reference 20 in main text]. Table 1 shows that 650 patients need to be added to the meta-analysis to reach statistical significance for the 45% increment of risk of SAEs reported by the UK-HARP-II trial (the only one reporting that endpoint for comparison of E/simvastatin vs. simvastatin alone).

| **Table A in S4 Appendix**  **Power analysis for SAEs in our main analysis** | | | |  |  |
| --- | --- | --- | --- | --- | --- |
| **Outcome** | **No. of trials** | **Risk ratio**  **(Fixed- effect- based)** | **P** | **Actual sample of meta-analysis** | **Sample that needs to be added to reach p<0.05 at power ≥80%** |
| SAEs | 1 | 1.45 | 0.087 | 203 | 650 |
