# Supplementary material for: Clinical Efficacy and Safety of Ezetimibe on Major Cardiovascular Endpoints: Systematic Review and Meta-Analysis of Randomized Controlled Trials
Source: PLoS One. 2015 Apr 27;10(4):e0124587. doi: 10.1371/journal.pone.0124587 (PMC4411142; doi:10.1371/journal.pone.0124587)
Supplement: S5 Appendix — (DOCX) [file pone.0124587.s006.docx]

**S5 Appendix - Missing Data Analysis**

For the arm-specific Absolute Risks calculations, for every trial, for every arm and for every outcome analyzed we used the *number of randomized patients* as denominator and the *number of events* reported as numerator. This is one of the most commonly used strategies [reference 21 in main text] and assumed in this particular instance that *none of the missing participants experienced the event*. Of course - like all methods when there are missing data - this analysis can be biased if the number of missing data is not small and the prognosis of patients not included in analyses differs between arms.

The medical literature presents uncertainty about the number of missing data sufficient to avoid a serious risk of bias, and very likely it differs in different trials. Sackett stated that a loss to follow-up of more than 20% can severely and irremediably distort any interpretation [Sackett DL, Rosenberg WM, Gray JA, Haynes RB, Richardson WS. Evidence basedmedicine: What it is and what it isn’t. BMJ. 1996; 312:71].

First, therefore, we analyzed the number of missing data in the trials included in our meta-analysis; second, we analyzed the reasons for their lack, if reported; third, we conducted a sensitivity **analysis to verify the robustness of our conclusions in alternative scenarios of missing data.**

**In the seven trials in our meta-analysis we detected 46 patients with missing data. That corresponds to 2.16% of missingness (46/2125). The reasons for the lack are illustrated in Table 1.**

We then compared our results with other types of analysis.

1. Available case analysis

We calculated the Absolute Risks of any outcome and any trial, inputting the number of non-missing patients as denominator and the number of observed events as numerator.

1. Input of missing data using reasons for missingness

We input - for any single trial’s missing data - values compatible with the reason for the lack, adopting the principles set out in the Cochrane Handbook [reference 16 in main text] and the analysis method illustrated by White [White IR, Higgins JPT. Meta-analysis with missing data In: Metaanalysis in Stata: an updated collection from the Stata Journal. Sterne JAC. 2009 Stata. Press ISBN-10:1-59718-049-1].

1. Gamble-Hollis analysis

For any RCT with missing binary data an *uncertainty interval* can be calculated, covering the confidence intervals across all possible allocations of the missing data in two extreme scenarios (extreme favoring intervention and extreme favoring control arm): then, from this *uncertainty interval* one can calculate *a new weight to assign to single trials,* toward a revised meta-analytical approach. Nevertheless, the Gamble-Hollis method too seems be “extreme”, because it can lead to too wide confidence intervals and considerable down-weighting of trials with missing data ~~[38~~][Gamble C and Hollis S Uncertainty method improved on best–worst case analysis in a binary meta-analysis Journal of Clinical Epidemiology 58 (2005)579–588 PMID:15878471].

The three approaches are illustrated in Tables 2-8, which report the meta-analysis results for all the outcomes and all patients. The three approaches are very similar, in terms of point estimates or confidence intervals. So we are confident that the small amount of missing data detected should not be associated with a real risk of biased results in our meta-analysis.

*Note that the small differences reported in Tables 2-8 for our analytical approach (= imputing zero) compared to the values that we gave for the general results of our meta-analysis (see text) are only due to the different method of pooling (Wolff inverse variance method versus Mantel-Haenszel method) used by the statistical package for sensitivity analysis for missing data (Stata command metamiss [37]), but this has no influence for the purpose of comparisons.*

| **Table A in S5 Appendix**Reasons for missing data for trials in our main analysis | | | | | | | |
| --- | --- | --- | --- | --- | --- | --- | --- |
| **Trial** | **Notes** | **Treatments (mg/day)** | **Allo-ca-ted (no.)** | **Miss-ing**  **(no.)** | **Reason for missingness** | **Assumed prognosis for missing patients** | **Missingdata manage-ment** |
| Arimura | The primary outcome was the angiographic change of coronary vessels | Interven-tion (E 10 +Atorva-statin 10) | 25 | 3 | Lost to follow-up | The same prognosis as in not missing subjects in the same arm | ICA-p |
|  |  | Control (Atorva-statin 10) | 25 | 1 | Lost to follow-up | The same prognosis as in not missing subjects in the same arm | ICA-p |
|  |  |  |  | 2 | Contra-indications | The same prognosis as in not missing subjects in the same arm | ICA-p |
| Kouvelos | The primary outcome was the first major CV event. The authors state that “all randomized patients completed the study protocol” | Interven-tion (E 10 +Rosuva-statin 10) | 126 | 0 | - |  | - |
|  |  | Control (Rosuva-statin10) | 136 | 0 | - |  | - |
| West | The primary outcome was the angiographic change in peripheral arterial atherosclerosis. Here we report as missing the *excluded cases* that appear not to have been follower ed until the end of the trials for safety outcomes considered in our meta-analysis. | Interven-tion (E 10+ simvastatin 40) | 22 | 1 | Lost to follow-up | The same prognosis as in not missing subjects in the same arm | ICA-p |
|  |  |  |  | 1 | Violation of inclusion criteria for clinically critical reasons | Have the outcome | ICA-1 |
|  |  |  |  | 1 | Clinical deteriora-tion | Have the outcome | ICA-1 |
|  |  | Control (simva-statin 40) | 22 | 3 | Lost to follow-up | The same prognosis as in not missing subjects in the same arm | ICA-p |
|  |  |  |  | 1 | Technical problems for detection of primary outcomes | The same prognosis as in not missing subjects in the same arm | ICA-p |
|  |  |  |  | 1 | Clinical deterioration | Have the outcome | ICA-1 |
| ENHANCE | The primary outcome was the angiographic change in carotid intima-media thickness; the authors state that all patients were included in the safety analysis but also that four patients were lost to follower -up | Interven-tion (E410 + Simvastatin 80) | 357 | 2 | Lost to follow-up | The same prognosis as in not missing subjects in the same arm | ICA-p |
|  |  | Control (Simva-statin 80) | 363 | 2 | Lost to follow-up | The same prognosis as in not missing subjects in the same arm | ICA-p |
| UK-HARP-II | The primary outcome was LDL-C lowering; the authors state that all randomized patients were included in the analysis. Table 4 indicates that the safety outcomes were reported for all randomized patients | Interven-tion (E10 + Simvastatin 20) | 101 | 0 | - | - | - |
|  |  | Control (Simva-statin 20)  ) | 102 | 0 | - | - | - |
| Ballantyne | The primary outcome was LDL-C lowering; the authors *explicitly* state that “discontinuations for withdrawal of consent, not compliance with protocol and lost to follow-up *were not different between groups*“, but do not show these data (p. 655). So we assumed zero for these cases, confident that the results were not biased in spite of a loss of statistical power (“missingness at random” situation). | Interven-tion (E 10 + Atorva-statin 10) | 201 | 0 | - | - | - |
|  |  | Control (Atorva-statin 10) | 45 | 0 | - | - | - |
| McKenney | The is an extension on 576 patients of a 12-week based trial(disease: Mixed Hyperlipidemia). The primary outcome was LDL-C lower ering; the efficacy analysis was stated as “all-patients-treated approach”; safety outcomes were reported as incidence rates per 1000 p-y for a difference in duration of exposure. The safety analysis indicates 229/340 subjects with almost one Adverse Event and 110/340 discontinu-ations in the intervention arm (145/236) and 149/236 in the control arm). We assumed (in spite of not being clearly stated by the Authors) that the not- compliant patients too were monitored for side effects until the end of the trial.  For our analysis we considered “lost to follow-up” only patients “who withdrew consent, moved, deviated from protocol, or were lost to follow-up”, whose number was reported. | Interven-tion (E10 + Fenofibrate 160) | 236 | 13 | Mixed reasons (withdrew consent, moved, deviated from protocol, Lost to follow-up) | The same prognosis as in not missing subjects in the same arm (the “no E” treatment in the control group is different from the “no E” treatment in the intervention group: the prognosis of not treated should also be different in the arms) | ICA-p |
|  |  | Control (Fenofib-rate 160) | 340 | 15 | Mixed reasons (withdrew consent, moved, deviated from protocol, lost to follow-up) | The same prognosis as in not missing subjects in the same arm (the “no E” treatment in the control group is different from the “no E” treatment in the intervention group: the prognosis of not treated should also be different in the arms) | ICA-p |
| ELIMIT | The primary outcome was the angiographic change in peripheral arterial atherosclerosis | Interven-tion (E 10 Simvastatin 40 Niacin 1500) | 42 | 0 | - | - | - |
|  |  | Control (Simva-statin 40) | 45 | 0 | - | - | - |
|  |  | Not specified |  | 1 | Refusal | The same prognosis as in not missing subjects in the same arm (?) | ICA-pc |
|  |  |  |  | 14 | Patient uncoopera-tive | The same prognosis as in not missing subjects in the same arm (?) | ICA-pc |

Notes

ICA-pc: the same probability of event as the control arm

ICA-p: the same probability of event as the same arm

ICA-1: input missing = 1

| **Table B in S5 Appendix** Missing data analysis: outcome CANCER | | | | | | |
| --- | --- | --- | --- | --- | --- | --- |
|  | Fixed effect-based | | | Random effect-based | | |
| Missing data management | RR | 95% CI  lower | 95% CI upper | RR | 95% CI lower | 95% CI upper |
| Inputting zero (our approach) |  | 2.379 | 0.28 | 20.201 | 2.146 | 0.075 |
| Ignoring missing data (available case analysis) |  | 2.379 | 0.28 | 20.201 | 2.146 | 0.075 |
| Inputting values according to clinical reasons |  | 2.379 | 0.28 | 20.201 | 2.146 | 0.075 |
| Gamble and Hollis approach (best-worst scenarios corrected) |  | 2.379 | 0.28 | 20.201 | 2.146 | 0.075 |

| **Table C in S5 Appendix** Missing data analysis: outcome DEATH | | | | | | |
| --- | --- | --- | --- | --- | --- | --- |
|  | Fixed effect-based | | | Random effect-based | | |
| Missing data management | RR | 95% CI lower | 95% CI upper | RR | 95% CI lower | 95% CI upper |
| Inputting zero (our approach) | 1.399 | 0.462 | 4.234 | 1.4 | 0.448 | 4.383 |
| Ignoring missing data (available case analysis) | 1.317 | 0.436 | 3.977 | 1.321 | 0.42 | 4.155 |
| Inputting values according to clinical reasons | 1.212 | 0.451 | 3.259 | 1.23 | 0.429 | 3.524 |
| Gamble and Hollis approach (best-worst scenarios corrected) | 1.017 | 0.293 | 3.529 | 1.017 | 0.293 | 3.529 |

| **Table D in S5 Appendix** Missing data analysis: outcome CV DEATH | | | | | | |
| --- | --- | --- | --- | --- | --- | --- |
|  | Fixed effect-based | | | Random effect-based | | |
| Missing data management | RR | 95% CI lower | 95% CI upper | RR | 95% CI lower | 95% CI upper |
| Inputting zero (our approach) | 1.3 | 0.318 | 5.314 | 1.297 | 0.239 | 7.04 |
| Ignoring missing data (available case analysis) | 1.301 | 0.318 | 5.315 | 1.298 | 0.239 | 7.04 |
| Inputting values according to clinical reasons | 1.301 | 0.318 | 5.315 | 1.298 | 0.239 | 7.04 |
| Gamble and Hollis approach (best-worst scenarios corrected) | 0.92 | 0.218 | 3.884 | 0.923 | 0.193 | 4.415 |

| **Table E in S5 Appendix** Missing data analysis: outcome NON-CV DEATH | | | | | | |
| --- | --- | --- | --- | --- | --- | --- |
|  | Fixed effect-based | | | Random effect-based | | |
| Missing data management | RR | 95% CI lower | 95% CI upper | RR | 95% CI lower | 95% CI upper |
| Inputting zero (our approach) | 1.045 | 0.109 | 9.969 | 1.045 | 0.109 | 9.969 |
| Ignoring missing data (available case analysis) | 1.045 | 0.109 | 9.969 | 1.045 | 0.109 | 9.969 |
| Inputting values according to clinical reasons | 1.045 | 0.109 | 9.969 | 1.045 | 0.109 | 9.969 |
| Gamble and Hollis approach (best-worst scenarios corrected) | 0.779 | 0.144 | 4.215 | 0.779 | 0.144 | 4.215 |

| **Table F in S5 Appendix** Missing data analysis: outcome MYOCARDIAL INFARCTION | | | | | | |
| --- | --- | --- | --- | --- | --- | --- |
|  | Fixed effect-based | | | Random effect-based | | |
| Missing data management | RR | 95% CI lower | 95% CI upper | RR | 95% CI lower | 95% CI upper |
| Inputting zero (our approach) | 1.356 | 0.343 | 5.354 | 1.356 | 0.343 | 5.354 |
| Ignoring missing data (available case analysis) | 1.296 | 0.328 | 5.112 | 1.296 | 0.328 | 5.112 |
| Inputting values according to clinical reasons | 1.375 | 0.395 | 4.783 | 1.375 | 0.395 | 4.783 |
| Gamble and Hollis approach (best-worst scenarios corrected) | 1.131 | 0.275 | 4.656 | 1.131 | 0.275 | 4.656 |

| **Table G in S5 Appendix** Missing data analysis: outcome STROKE | | | | | | |
| --- | --- | --- | --- | --- | --- | --- |
|  | Fixed effect-based | | | Random effect-based | | |
| Missing data management | RR | 95% CI lower | 95% CI upper | RR | 95% CI lower | 95% CI upper |
| Inputting zero (our approach) | 1.349 | 0.363 | 5.006 | 1.349 | 0.363 | 5.006 |
| Ignoring missing data (available case analysis) | 1.292 | 0.349 | 4.789 | 1.292 | 0.349 | 4.789 |
| Inputting values diversified by clinical reasons | 1.393 | 0.421 | 4.609 | 1.393 | 0.421 | 4.609 |
| Gamble and Hollis approach (best-worst scenarios corrected) | 1.359 | 0.316 | 5.848 | 1.359 | 0.316 | 5.848 |

| **Table H in S5 Appendix** Missing data analysis: outcome SAEs | | | | | | |
| --- | --- | --- | --- | --- | --- | --- |
|  | Fixed effect-based | | | Random effect-based | | |
| Missing data management | RR | 95% CI lower | 95% CI upper | RR | 95% CI lower | 95% CI upper |
| Inputting zero (our approach) | 1.256 | 0.899 | 1.754 | 1.256 | 0.899 | 1.754 |
| Ignoring missing data (available case analysis) | 1.246 | 0.893 | 1.741 | 1.246 | 0.893 | 1.741 |
| Inputting values diversified by clinical reasons | 1.246 | 0.893 | 1.741 | 1.246 | 0.893 | 1.741 |
| Gamble and Hollis approach (best-worst scenarios corrected) | 1.28 | 0.884 | 1.853 | 1.28 | 0.884 | 1.853 |
