# Supplementary material for: Clinical Efficacy and Safety of Ezetimibe on Major Cardiovascular Endpoints: Systematic Review and Meta-Analysis of Randomized Controlled Trials
Source: PLoS One. 2015 Apr 27;10(4):e0124587. doi: 10.1371/journal.pone.0124587 (PMC4411142; doi:10.1371/journal.pone.0124587)
Supplement: S6 Appendix — (DOCX) [file pone.0124587.s007.docx]

**S6 Appendix**

**Table A in S6 Appendix: List of trials included (main analysis)**

| Trial | Year | Authors | Title | Reference |
| --- | --- | --- | --- | --- |
| Arimura | 2012 | Arimura T, Miura SI, Ike A, Sugihara M, Iwata A, Nishikawa H, Kawamura A, Saku K | Comparison of the efficacy and safety of statin and statin/ezetimibe therapy after  coronary stent implantation in patients with stable angina | J Cardiol. 2012 Aug;60(2):111-8 [PMID: 22542530] |
| Kouvelos | 2013 | Kouvelos GN, Arnaoutoglou EM, Matsagkas MI, Kostara C, Gartzonika C, Bairaktari ET, Milionis HJ | Effects of rosuvastatin with or without ezetimibe on clinical outcomes in patients undergoing elective vascular surgery: results of a pilot study | J Cardiovasc Pharmacol Ther. 2013 Jan;18(1):5-12 [PMID: 22573476] |
| ENHANCE | 2008 | Kastelein JJ, Akdim F, Stroes ES, Zwinderman AH, Bots ML, et al. ENHANCE Investigators | Simvastatin with or without ezetimibe in familial hypercholesterolemia | N Engl J Med. 2008 Apr 3;358(14):1431-43 [PMID: 18376000 ] |
| UK-HARP-II | 2006 | Landray M, Baigent C, Leaper C, Adu D, Altmann P, et al. | The second United Kingdom Heart and Renal Protection (UK-HARP-II) Study: a randomized controlled study of the biochemical safety and efficacy of adding ezetimibe to simvastatin as initial therapy among patients with CKD | Am J Kidney Dis. 2006 Mar;47(3):385-95 [PMID: 16490616] |
| Ballantyne | 2004 | Ballantyne CM, Lipka LJ, Sager PT, Strony J, Alizadeh J, Suresh R, Veltri EP | Long-term safety and tolerability profile of ezetimibe and atorvastatin coadministration therapy in patients with primary hypercholesterolaemia | Int J Clin Pract. 2004 Jul;58(7):653-8b [PMID: 15311720] |
| West | 2011 | West AM, Anderson JD, Epstein FH, Meyer CH, Wang H, et al. | Low-density lipoprotein lowering does not improve calf muscle perfusion, energetics, or exercise performance in peripheral arterial disease | J Am Coll Cardiol. 2011 Aug 30;58(10):1068-76 [PMID: 21867844] |
| McKenney | 2006 | McKenney JM, Farnier M, Lo KW, Bays HE, Perevozkaya I, Carlson G, Davies MJ, Mitchel YB, Gumbiner B | Safety and efficacy of long-term co-administration of fenofibrate and ezetimibe in patients with mixed hyperlipidemia | J Am Coll Cardiol. 2006 Apr 18;47(8):1584-7 [PMID: 16630994] |

**Table B in S6 Appendix: List of trials included (complementary analysis)**

| Baigent C et al | 2011 | Baigent C, Landray MJ, Reith C, Emberson J, Wheeler DC, et al. On behalf of the SHARP Investigators* | SHARP Investigators. The effects of lowering LDL cholesterol with simvastatin plus ezetimibe in patients with chronic kidney disease (Study of Heart and Renal Protection): a randomised placebo-controlled trial | Lancet. 2011 Jun 25;377(9784):2181-92 [PMID: 21663949] |
| --- | --- | --- | --- | --- |
| Rossebø AB et al | 2008 | Rossebø AB, Pedersen TR,  Boman K, Brudi P, et al. For the SEAS Investigators* | SEAS Investigators Intensive lipid lowering with simvastatin and ezetimibe in aortic stenosis | N Engl J Med. 2008 Sep 25;359(13):1343-56 [PMID: 18765433] |

**Table C in S6 Appendix: List of trials excluded**

| **First Author** | **Title** | **Reference** | **Reasons for exclusion** | | | |
| --- | --- | --- | --- | --- | --- | --- |
|  |  |  | **Design** | **Duration** | **Data problems** | **Other** |
| Abate N | Effect of ezetimibe/simvastatin versus atorvastatin or rosuvastatin on modifying lipid profiles in patients with diabetes, metabolic syndrome, or neither: Results of two subgroup analyses. | J Clin Lipidol. 2008 Apr;2(2):91-105. doi: 10.1016/j.jacl.2008.02.002. Epub 2008 Feb 15 |  | 6 weeks |  |  |
| AIM-HIGH Investigators | The Role of Niacin in Raising HDL-C to Reduce Cardiovascular Events in Patients with Atherosclerotic Cardiovascular Disease and Optimally Treated LDL-C AIM-HIGH: Rationale and Study Design* | Am Heart J. 2011 March ; 161(3): 471–477 |  |  |  | Not ezetimibe trial |
| AIM-HIGH Investigators | Niacin in Patients with Low HDL Cholesterol Levels Receiving Intensive Statin Therapy | N Engl J Med 2011;365:2255-67. |  |  |  | Not ezetimibe trial |
| Alvarez-Sala LA | Effects of fluvastatin extended-release (80 mg) alone and in combination with ezetimibe (10 mg) on low-density lipoprotein cholesterol and inflammatory parameters in patients with primary hypercholesterolemia: A 12-week, multicenter, randomized, open-label, parallel-group study | Clinical therapeutics 2008 30 1 84 Alvarez-Sala LA |  | 12 weeks |  |  |
| Araujo DB | Pleiotropic effects with equivalent low-density lipoprotein cholesterol reduction: comparative study between simvastatin and simvastatin/ezetimibe coadministration | Journal of cardiovascular pharmacology 2010 55 1 1 Araujo DB JCardiovascPharmacol 2010 55:1-5 |  | 6 weeks |  |  |
| Assmann G | Effects of ezetimibe, simvastatin, atorvastatin, and ezetimibe -statin therapies on non-cholesterol sterols in patients with primary hypercholesterolemia | Current Medical Research and Opinion 2008 24:1 (249-259) |  |  |  | Not ezetimibe trial |
| Averna M | Lipid-altering efficacy of switching to ezetimibe/simvastatin 10/20 mg versus rosuvastatin 10 mg in high-risk patients with and without metabolic syndrome | Diabetes and Vascular Disease Research 2011 8 262 Averna |  | 6 weeks |  |  |
| Averna M | Ezetimibe/simvastatin 10/20 mg versus simvastatin 40mg in coronary heart disease patients | Journal of clinical lipidology 2010 4 4 272 Averna M |  | 6 weeks |  |  |
| Azar M | Comparison of the Effects of Combination Atorvastatin (40 mg) + Ezetimibe (10 mg) Versus Atorvastatin (40 mg) Alone on Secretory Phospholipase A2 Activity in Patients With Stable Coronary Artery Disease or Coronary Artery Disease Equivalent | The American journal of cardiology 2011 107 11 1571 Azar M |  | 8 weeks |  |  |
| Ballantyne | Effect of Ezetimibe Coadministered With Atorvastatin in 628 Patients With Primary Hypercholesterolemia: A Prospective, Randomized, Double-Blind Trial | Circulation-2003-Ballantyne-2409-15 |  | 12 weeks |  |  |
| Ballantyne C.M | Efficacy and safety of ezetimibe co-administered with simvastatin compared with atorvastatin in adults with hypercholesterolemia | American Journal of Cardiology 2004 93:12 (1487-1494 |  |  | No outcome data |  |
| Ballantyne C.M. | 21st-century treatment of dyslipidemia: The potential role of cholesterol absorption inhibitors in combination therapy Ballantyne C.M. | Advanced Studies in Medicine 2003 3:4 C (S324-S328) | not RCT |  |  |  |
| Ballantyne CM, et al | Efficacy and safety of rosuvastatin 40 mg alone or in combination with ezetimibe in patients at high risk of cardiovascular disease (results from the EXPLORER study). | Am J Cardiol. 2007 Mar 1;99(5):673-80. Epub 2007 Jan 4. |  | 6 weeks |  |  |
| Bardini G | Ezetimibe + simvastatin versus doubling the dose of simvastatin in high cardiovascular risk diabetics: a multicenter, randomized trial (the LEAD study) | Cardiovascular Diabetology 2010 9 20 Bardini |  | 6 weeks |  |  |
| Barrios V | Lipid-altering efficacy of switching from atorvastatin 10 mg/day to ezetimibe/simvastatin 10/20 mg/day compared to doubling the dose of atorvastatin in hypercholesterolaemic patients with atherosclerosis or coronary heart disease | International journal of clinical practice 2005 59 12 1377 Barrios V |  | 6 weeks |  |  |
| Baruch L | Effect of serum lipid levels of switching dose of ezetimibe from 10 to 5 mg | The American journal of cardiology 2009 103 11 1568 Baruch L |  | 4 weeks |  |  |
| Baruch L | Efficacy of ezetimibe 2.5 mg with a novel tablet-splitting strategy | American Journal of Pharmacy Benefits 2010 2:4 (261-266) |  | 6 weeks |  |  |
| Bays HE | Effectiveness and tolerability of ezetimibe in patients with primary hypercholesterolemia : pooled analysis of two phase II studies | Clinical therapeutics 2001 23 8 1209 Bays HE |  | 12 weeks |  |  |
| Bays HE | A multicenter, randomized, double-blind, placebo-controlled, factorial design study to evaluate the lipid-altering efficacy and safety profile of the ezetimibe/simvastatin tablet compared with ezetimibe and simvastatin monotherapy in patients with primary hypercholesterolemia | Clinical therapeutics 2004 26 11 1758 Bays HE |  | 12 weeks |  |  |
| Bays HE | Effects of coadministered ezetimibe plus fenofibrate in mixed dyslipidemic patients with metabolic syndrome | Metabolic syndrome and related disorders 2011 9 2 135 Bays HE |  | 12 weeks |  |  |
| Bays HE | Safety and Efficacy of Ezetimibe Added on to Rosuvastatin 5 or 10 mg Versus Up-Titration of Rosuvastatin in Patients With Hypercholesterolemia (the ACTE Study) | The American journal of cardiology 2011 108 4 523 Bays HE |  | 6 weeks |  |  |
| Bays HE, et al | Efficacy and safety of ezetimibe added to atorvastatin versus atorvastatin uptitration or switching to rosuvastatin in patients with primary hypercholesterolemia | Am J Cardiol. 2013 Dec 15;112(12):1885-95. doi: 10.1016/j.amjcard.2013.08.031. Epub 2013 Sep 21. |  | 12 weeks |  |  |
| Ben-Yehuda O et al | The comparative efficacy of ezetimibe added to atorvastatin 10 mg versus uptitration to atorvastatin 40 mg in subgroups of patients aged 65 to 74 years or greater than or equal to 75 years. | J Geriatr Cardiol. 2011;8:1-11. [PMID: 22783278] |  | 12 weeks |  |  |
| Bergman AJ et al | Interaction of single-dose ezetimibe and steady-state cyclosporine in renal transplant patients | J Clin Pharmacol. 2006 Mar;46(3):328-36. | not RCT |  |  |  |
| Bergman AJ, et al | Effects of ezetimibe on cyclosporine pharmacokinetics in healthy subjects. | J Clin Pharmacol. 2006 Mar;46(3):321-7. |  | 2 weeks |  |  |
| Berneis K, et al | Ezetimibe alone or in combination with simvastatin increases small dense low-density lipoproteins in healthy men: a randomized trial. | Eur Heart J. 2010 Jul;31(13):1633-9. Jul;31(13):1633-9. |  | 2 weeks |  |  |
| Berthold HK | Therapie der familiaren Hypercholesterinamie mit oder ohne ezetimib | Internist 2008 19:1274-1276 |  |  |  | ENHANCE 2008 data |
| Berthold HK | Effect of ezetimibe and/or Simvastatin on Coenzyme Q10 levels in plasma | Drug safety an international journal of medical toxicology and drug experience 2006 29 8 703 Berthold HK |  | 2 weeks |  |  |
| Berthold HK | Evidence from a Randomized Trial That Simvastatin, but Not Ezetimibe, Upregulates Circulating PCSK9 Levels | PlosOne 2013 8 e60095 Berthold |  | 12 weeks |  |  |
| Bogiatzi C et al | Ezetimibe and Regression of Carotid Atherosclerosis: Importance of Measuring Plaque David Spence Burden | Stroke. 2012;43:1153-1155; originally |  |  | no outcome data |  |
| Brohet C | LDL-C goal attainment with the addition of ezetimibe to ongoing simvastatin treatment in coronary heart disease patients with hypercholesterolemia | Curr Med Res Opin 2005 21 571-8 Brohet |  | 6 weeks |  |  |
| Brunner G, ET AL | The Effect of Lipid Modification on Peripheral Artery Disease after Endovascular Intervention Trial (ELIMIT). | Atherosclerosis. 2013 Dec;231(2):371-7 [PMID: 24267254] |  |  |  | inadequate comparison (not possible to evaluate the net effect of E) |
| Catapano AL, et al. | Lipid-altering efficacy of the ezetimibe/simvastatin single tablet versus rosuvastatin in hypercholesterolemic patients. | Curr Med Res Opin 2006;22:2041–53. |  | 6 weeks |  |  |
| Chenot F | Co-administration of ezetimibe and simvastatin in acute myocardial infarction | European journal of clinical investigation 2007 37 5 357 Chenot F |  | 1 week |  |  |
| Cho YK, Hur SH, Han CD, Park HS, Yoon HJ, Kim H, et al. | Comparison of Ezetimibe/Simvastatin 10/20 mg Versus Atorvastatin 20 mg in Achieving a Target Low Density Lipoprotein-Cholesterol Goal for Patients With Very High Risk. | Korean Circ J. 2011;41:149-53. [PMID: 21519514] |  | 6 weeks |  |  |
| Chow D | Short-term ezetimibe is well tolerated and effective in combination with statin therapy to treat elevated LDL cholesterol in HIV-infected patients | AIDS 2009 23 2133–41 Chow |  | 12weeks |  |  |
| Clarenbach JJ | The lipid-lowering effect of ezetimibe in pure vegetarians | J. Lipid Res.-2006-Clarenbach-2820-4 |  | 2 weeks |  |  |
| Coll B | Ezetimibe effectively decreses LDL-cholesterol in HIV-infected patients | AIDS (London, England) 2006 20 12 1675 Coll B |  | 6 weeks |  |  |
| Conard SE | Efficacy and safety of ezetimibe added on to atorvastatin (20 mg) versus uptitration of atorvastatin (to 40 mg) in hypercholesterolemic patients at moderately high risk for coronary heart diseas | The American journal of cardiology 2008 102 11 1489 Conard SE |  | 6 weeks |  |  |
| Constance C, et al | Efficacy of ezetimibe/simvastatin 10/20 and 10/40 mg compared with atorvastatin 20 mg in patients with type 2 diabetes mellitus. | Diabetes Obes Metab. 2007;9:575-84. [PMID: 17451425] |  | 6 weeks |  |  |
| Cruz-Fernandez JM | Efficacy and safety of ezetimibe co-administered with ongoing atorvastatin therapy in achieving low-density lipoprotein goal in patients with hypercholesterolemia and coronary heart disease | International journal of clinical practice 2005 59 6 619 Cruz-Fernández JM |  | 6 weeks |  |  |
| Davidson MH, et al | Efficacy and safety of ezetimibe coadministered with statins: randomised, placebo-controlled, blinded experience in 2382 patients with primary hypercholesterolemia. | Int J Clin Pract 2004;58:746–55. |  | 12 weeks |  |  |
| Davidson MH, et al. | Ezetimibe coadministered with simvastatin in patients with primary hypercholesterolemia. | J Am Coll Cardiol 2002;40:2125–34 |  | 12 weeks |  |  |
| Denke M | Ezetimibe added to ongoing statin therapy improves LDL-C goal attainment and lipid profile in patients with diabetes or metabolic syndrome | Diab Vasc Dis Res 2006 Denke M Sep 3 2 93-102 |  | 6 weeks |  |  |
| Derosa G | Efficacy and safety of ezetimibe/simvastatin association in non-diabetic and diabetic patients with polygenic hypercholesterolemia or combined hyperlipidemia and previously intolerant to standard statin treatment | Journal of clinical pharmacy ant therapeutics 2009 34 267-276 | not RCT |  |  |  |
| Dujovne C.A | Safety and efficacy of ezetimibe monotherapy in 1624 primary hypercholesterolaemic patients for up to 2 years | International Journal of Clinical Practice 2008 62:9 (1332-1336) | not RCT |  |  |  |
| Dujvone CA | Efficacy and safety of a potent new selective cholesterol absorption inhibitor, ezetimibe, in patients with primary hypercholesterolemia | The American journal of cardiology 2002 90 10 1092 Dujovne CA |  | 12 weeks |  |  |
| Ezzet F | The plasma concentration and LDL-C relationship in patients receiving ezetimibe | Journal of clinical pharmacology 2001 41 9 943 Ezzet F |  | 12 weeks |  |  |
| Farmer J. | The Vytorin on Carotid-Media Thickness and Overall Arterial Rigidity (VYCTOR) study | Expert Review of Cardiovascular Therapy 2009 7:9 (1057-1060) |  |  |  | Comment ?? |
| Farnier M | Comparative efficacy and safety of fenofibrate/pravastatin plus ezetimibe triple therapy and simvastatin/ezetimibe dual therapy in type 2 diabetic patients with mixed hyperlipidaemia and cardiovascular disease | Diabetes and Vascular Disease Research 2012 9 205 Farnier |  | 12 weeks |  |  |
| Farnier M | Lipid-altering efficacy of ezetimibe⁄simvastatin 10⁄20 mg compared with rosuvastatin 10 mg in high-risk hypercholesterolaemic patients inadequately controlled with prior statin monotherapy – The IN-CROSS study | Int J Clin Pract 2009 Fernier M Apr 63 4 547-59 |  | 6 weeks |  |  |
| Farnier M | Effect of co-administering ezetimibe with on-going simvastatin treatment on LDL-C goal attainment in hypercholesterolemic patients with coronary heart disease | Inter J Cardiol 2005 102 2 327 Farnier M |  | 6 weeks |  |  |
| Farnier M, et al | Efficacy and safety of the coadministration of ezetimibe with fenofibrate in patients with mixed hyperlipidaemia. | Eur Heart J. 2005 May;26(9):897-905. Epub 2005 Mar 21. |  | 12 weeks |  |  |
| Farnier M, Roth E, Gil-Extremera B, et al. | Ezetimibe/Simvastatin þ Fenofibrate Study Group. Efficacy and safety of the coadministration of ezetimibe/simvastatin with fenofibrate in patients with mixed hyperlipidemia. | Am Heart J. 2007;153:335.e1-8. |  | 12 weeks |  |  |
| Fazio S | Long-term efficacy and safety of ezetimibe/simvastatin coadministered with extended-release niacin in hyperlipidaemic patients with diabetes or metabolic syndrome | Diabetes, obesity & metabolism 2010 12 11 983 Fazio S |  |  |  | Subgroup analysis Guyton Study |
| Fazio S | Long-term safety and efficacy of triple combination ezetimibe/simvastatin plus extended-release niacin in patients with hyperlipidemia | The American journal of cardiology 2010 105 4 487  Fazio S |  |  |  | Same patients as Guyton Study |
| Feldman T, et al | Efficacy and safety of ezetimibe/simvastatin versus simvastatin monotherapy in hypercholesterolemic patients with metabolic syndrome. | Metab Syndr Relat Disord. 2007 Spring;5(1):13-21. doi: 10.1089/met.2006.0033. |  | 12 weeks |  |  |
| Feldman T, et al | Treatment Of High-Risk Patients With Ezetimibe Plus Simvastatin Co-Administration Versus Simvastatin Alone To Attain National Cholesterol Education Program Adult Treatment Panel III Low- Density Lipoprotein Cholesterol Goals | Am J Cardiol 2004 93:1481-1486 |  | 23 weeks |  |  |
| Fichtlscherer S, et al | Differential effects of short-term lipid lowering with ezetimibe and statins on endothelial function in patients with CAD: clinical evidence for 'pleiotropic' functions of statin therap | Eur Heart J. 2006 May;27(10):1182-90. Epub 2006 Apr 18. | not RCT |  |  |  |
| Fleg JL et al | Effect of Statins Alone Versus Statins Plus  Ezetimibe on Carotid Atherosclerosis in Type 2 Diabetes  The SANDS (Stop Atherosclerosis in Native Diabetics Study) Trial | Journal of the American College of Cardiology  2008 Dec 16;52(25):2198-205 PMID: 19095139] |  |  |  | Type/dosage of comparator not evaluable |
| Florentin M | The effect of simvastatin alone versus simvastatin plus ezetimibe on the concentration of small dense low-density lipoprotein cholesterol in subjects with primary hypercholesterolemia | CMRO 2011 Florentin M 27 3 685-92 |  | 3 months |  |  |
| Foody JM, , et al. | Safety and efficacy of ezetimibe/simvastatin combination versus atorvastatin alone in adults 65 years of age with hypercholesterolemia and with or at moderately high/high risk for coronary heart disease (the VYTELD study). | Am J Cardiol. 2010;106:1255-63. [PMID: 21029821] |  | 12 weeks |  |  |
| Gagne C | Efficacy and safety of ezetimibe coadministered with atorvastatin or simvastatin in patients with homozygous familial hypercholesterolemia | Circulation-2002-Gagné-2469-75 |  | 12 weeks |  |  |
| Gagne C | Efficacy and safety of ezetimibe added to ongoing statin therapy for treatment of patients with primary hypercholesterolemia | The American journal of cardiology 2002 90 10 1084 Gagné C |  | 8 weeks |  |  |
| Gaudiani LM et al | Efficacy and safety of ezetimibe co-administered with simvastatin in thiazolidinedione-treated type 2 diabetic patients | Diabetes Obes Metab. 2005 Jan;7(1):88-97 [PMID: 15642080] |  |  |  | Different doses of statin in the arms |
| Geiss HC | Effects of ezetimibe on plasma lipoproteins in severely hypercholesterolemic patients treated with regular LDL-apheresis and statins | Atherosclerosis 2005 180 1 107 Geiss HC |  | 5 weeks |  |  |
| Geiss HC | Effect of ezetimibe on low-density lipoprotein subtype distribution: results of a placebo-controlled, double-blind trial in patients treated by regular low-density lipoprotein apheresis and statins | Metabolism clinical and experimental 2006 55 5 599 Geiss HC |  | 5 weeks |  |  |
| Goldberg AC | Efficacy and Safety of Ezetimibe Coadministered With Simvastatin in Patients With Primary Hypercholesterolemia: A Randomized, Double-Blind, Placebo-Controlled Trial | Mayo Clinic proceedings. Mayo Clinic 2004 79 5 620 Goldberg AC |  | 12 weeks |  |  |
| Goldberg RB | Ezetimibe/Simvastatin vs Atorvastatin in Patients With Type 2 Diabetes Mellitus and Hypercholesterolemia: The VYTAL Study | Mayo Clinic proceedings. Mayo Clinic 2006 81 12 1579 Goldberg RB |  | 6 weeks |  |  |
| Gonzalez-Ortiz M | Effect of ezetimibe on insulin sensitivity and lipid profile in obese and dyslipidaemic patients | Cardiovascular drugs and therapy 2006 20 2 143 González-Ortiz M |  | 3 months |  |  |
| Gouni-Berthold I | Effects of ezetimibe and/or simvastatin on LDL receptor protein expression and on LDL receptor and HMG-CoA reductase gene expression: A randomized trial in healthy men | Atherosclerosis 2008 198 198-207 Gouni-Berthold |  | 2 weeks |  |  |
| Guyton JR, et al | Lipoprotein and apolipoprotein ratios in the VYTAL trial of ezetimibe/ simvastatin compared with atorvastatin in type 2 diabetes. | J Clin Lipidol. 2008; 2:19-24. [PMID: 21291711] |  | 6 weeks |  |  |
| Guyton JR, et al | Lipid-Altering Efficacy and  Safety of Ezetimibe/Simvastatin  Coadministered With Extended-Release Niacin  in Patients With Type IIa or Type IIb Hyperlipidemia | J Am Coll Cardiol. 2008 Apr 22;51(16):1564-72 [PMID: 18420099] |  |  |  | inadequate comparison (not possible to assess the net effect of E) |
| Hajer GRet al | Lipid-lowering therapy does not affect the postprandial drop in high density lipoprotein-cholesterol (HDL-c) plasma levels in obese men with metabolic syndrome: a randomized |  |  | 6 weeks |  |  |
| Hamdan R, et al | Benefit and tolerability of the coadministration of ezetimibe and atorvastatin in acute coronary syndrome patients. | .J Med Liban. 2011 Apr-Jun;59(2):65-9. |  | 12 weeks |  |  |
| Hamilton-Craig I., | At Sea with SEAS: The First Clinical Endpoint Trial for Ezetimibe, Treatment of Patients with Mild to Moderate Aortic Stenosis, Ends with Mixed Results and More Controversy | Heart Lung and Circulation 2009 18:5 (343-346) |  |  |  | Comment to SEAS trial |
| Her Ae-Young | Effects of atorvastatin 20 mg, rosuvastatin 10 mg, and atorvastatin/ezetimibe 5 mg/5 mg on lipoproteins and glucose metabolism. | Journal of cardiovascular pharmacology and therapeutics 2010 15 2 167 Her AY |  | 8 weeks |  |  |
| Ichimori s | Ezetimibe improves glucose metabolism by ameliorating hepatic function in Japanese patients with type 2 diabetes | J Diabetes Invest, doi: 10.1111/j.2040-1124.2011.00147.x, 2012 | not RCT |  |  |  |
| Inazawa t | RESEARCH (Recognized effect of Statin and ezetimibe therapy for achieving LDL-C Goal), a randomized, doctor-oriented, multicenter trial to compare the effects of higher-dose statin versus ezetimibe-plus-statin on the serum LDL-C concentration of Japanese type-2 diabetes patients design and rationale | Lipids in Health and Disease 2013, 12:142 |  |  | No outcome data |  |
| Jakulj L | Baseline cholesterol absorption and the response to ezetimibe/simvastatin therapy: a post-hoc analysis of the ENHANCE trial | J Lipid Res 2010 51 755–762 Jakuli |  |  | No outcome data | Post hoc analysis ENHANCE data |
| Jara EG | Efectos del bezafibrato combinado con simvastatina o con ezetimibe en la función endotelial y el perfil de lípidos de pacientes hipertensos con hipertrigliceridemia, hipoalfalipoproteinemia y colesterol-LDL limítrofe | Med Int Mex 2007;23(5):371-5 |  | 4 weeks |  |  |
| Jimenez JG, et al | The efficacy and safety of ezetimibe/simvastatin combination compared with intensified lipid-lowering treatment strategies in diabetic subjects with and without metabolic syndrome | Diabetes Obes Metab. 2013;15:513-22. [PMID: 23279632] |  | 6 weeks |  |  |
| Kanat M | A multi-center, open label, crossover designed prospective study evaluating the effects of lipid lowering treatment on steroid synthesis in patients with Type 2 diabetes (MODEST Study). | Journal of endocrinological investigation, 2009, 32(10), 852 |  |  | No outcome data |  |
| Kawagoe Y | Comparative study between high-dose fluvastatin and low-dose fluvastatin and ezetimibe with regard to the effect on endothelial function in diabetic patients | Endocr J 2011 58 171-5 Kawagoe |  | 10 weeks |  |  |
| Kawashiri MA | Efficacy and safety of coadministration of rosuvastatin, ezetimibe , and colestimide in heterozygous familial hypercholesterolemia. | The American journal of cardiology, 2012, 109(3), 364 |  |  |  | Not ezetimibe trial |
| Kerzner B | Efficacy and safety of ezetimibe coadmistered with lovastatin in primary hypercholesterolemia | The American journal of cardiology 2003 91 4 418 Kerzner B |  | 12 weeks |  |  |
| Kinouchi K | Effects of adding Ezetimibe to Fluvastatin on kidney function in patients with hypercholesterolemia: a randomized control trial | Journal of atherosclerosis and thrombosis 20(3):245 |  |  | No outcome data |  |
| Knopp RH | Effects of ezetimibe, a new cholesterol absorption inhibitor, on plasma lipids in patients with primary hypercholesterolemia | EurHeart J 24:729-741 2003 |  | 12 weeks |  |  |
| Knopp RH | Evaluation of the efficacy, safety, and tolerability of ezetimibe in primary hypercholesterolaemia: a pooled analysis from two controlled phase III clinical studies | Int J clin pract 2003 Knopp RH 363 |  | 12 weeks |  |  |
| Knopp RH | Lipoprotein effects of combined ezetimibe and colesevelam hydrochloride versus ezetimibe alone in hypercholesterolemic subjects: a pilot study | Metabolism clinical and experimental 2006 55 12 1697 Knopp RH |  | 12 weeks |  |  |
| Konstandin MH, et al | Ezetimibe effectively lowers LDL-cholesterol in cardiac allograft recipients on stable statin therapy. | Clin Transplant. 2008 Sep-Oct;22(5):639-44. doi: 10.1111/j.1399-0012.2008.00838.x. Epub 2008 May 19. | not RCT |  |  |  |
| Kosoglou T | Pharmacodynamic interaction between the new selective cholesterol absorption inhibitor ezetimibe and simvastatin | Br J Clin Pharmacol 2002 Kosoglou T Sep 54 3 309-19 |  | 2 weeks |  |  |
| Kosoglou TK | Effects of ezetimibe on the pharmacodynamics and pharmacokinetics of lovastatin | Curr Med Res Opin 2004 20 955-65 Kosoglou |  | 2 weeks |  |  |
| Kovarnik T | Virtual Histology Evaluation of Atherosclerosis Regression During Atorvastatin and Ezetimibe Administration – HEAVEN Study – | Circ J 2012; 76: 176 – 183) |  |  | No outcome data |  |
| Kumar SS | Comparison of the efficacy of administering a combination of ezetimibe plus fenofibrate versus atorvastatin monotherapy in the treatment of dyslipidemia | Lipids in Health and Disease 2009 8 56 Kumar |  | 6 weeks |  |  |
| Lakoski SG | Indices of Cholesterol Metabolism and Relative Responsiveness to Ezetimibe and Simvastatin | J Clin Endocrinol Metab 2010 95 800-9 Lakoski |  | 6 weeks |  |  |
| Lee JH, et al | Effects of ezetimibe/simvastatin 10/20 mg vs. atorvastatin 20 mg on apolipoprotein B/apolipoprotein A1 in Korean patients with type 2 diabetes mellitus: results of a randomized controlled trial. | Am J Cardiovasc Drugs. 2013;13:343-51. [PMID:23728830] |  | 12 weeks |  |  |
| Lee Sang-Hak | The Effects of Statin Monotherapy and Low-Dose Statin/Ezetimibe on Lipoprotein-Associated Phospholipase A2 | Clin Cardiol 2011 Lee SH Feb 34 2 108-12 |  | 8 weeks |  |  |
| Leiter LA | Efficacy and safety of ezetimibe added on to atorvastatin (40 mg) compared with uptitration of atorvastatin (to 80 mg) in hypercholesterolemic patients at high risk of coronary heart disease | The American journal of cardiology 2008 102 11 1495 Leiter LA |  | 12 weeks |  |  |
| Leiter LA et al | Attainment of Canadian and European guidelines lipids targets with atorvastatin plus ezetimibe vs doubling the dose of atorvastatin | Int J Clin Pract 2010 64 1765 |  | 6 weeks |  |  |
| Lin X | Combined effects of ezetimibe and phytosterols on cholesterol metabolism: a randomized, controlled feeding study in humans. | Circulation 2011 124 596-601 Lin |  | 3 weeks |  |  |
| Lipka L | Efficacy and safety of coadministration of ezetimibe and statins in elderly patients with primary hypercholesterolaemia | Drugs & aging 2004 21 15 1025 Lipka L |  | 12 weeks |  |  |
| Liu J | Comparative study of high-dose Xuezhikang and low-dose Xuezhikang plus ezetimibe on pro- and anti-inflammatory markers | Clinical Lipidology 2013 8:1 (151-157) |  | 6 weeks |  |  |
| Liu Ping-Yen | Evidence for Statin Pleiotropy in Humans: Differential Effects of Statins and Ezetimibe on Rho-Associated Coiled-Coil Containing Protein Kinase Activity, Endothelial Function, and Inflammation | Circulation-2009-Liu-131-8 |  | 4 weeks |  |  |
| Malmstrom RE, et al | No effect of lipid lowering on platelet activity in patients with coronary artery disease and type 2 diabetes or impaired glucose tolerance. | Thromb Haemost 2009;101: 157-64. |  | 6 weeks |  |  |
| Maron DJ | Impact of adding ezetimibe to statin to achieve low-density lipoprotein cholesterol goal (from the Clinical Outcomes Utilizing Revascularization and Aggressive Drug Evaluation [COURAGE] trial) | The American journal of cardiology 2013 111 11 1557 Maron DJ |  |  | No outcome data |  |
| Masana L et al |  | Ezetimibe Study Group Long-term safety and, tolerability profiles and lipid-modifying efficacy of ezetimibe coadministered with ongoing simvastatin treatment: a multicenter, randomized, double-blind, placebo-controlled, 48-week extension study | Clin Ther. 2005 Feb;27(2):174-84 [PMID: 15811480] |  |  | Type/dosage of comparator not evaluable |
| Matsue Y, et al | Differences in action of atorvastatin and ezetimibe in lowering low-density lipoprotein cholesterol and effect on endothelial function: randomized controlled trial. | Circ J. 2013;77(7):1791-8. Epub 2013 Apr 19. |  | 12 weeks |  |  |
| McCormack T | Incremental cholesterol reduction with ezetimibe/simvastatin, atorvastatin and rosuvastatin in UK General Practice (IN-PRACTICE): randomised controlled trial of achievement of Joint British Societies (JBS-2) cholesterol targets | International journal of clinical practice 2010 64 8 1052 McCormack T |  | 6 weeks |  |  |
| McKenney J | LDL-C Goal Attainment With Ezetimibe Plus Simvastatin Coadministration vs Atorvastatin or Simvastatin Monotherapy in Patients at High Risk of CHD | LDL-C Goal Attainment With Ezetimibe Plus Simvastatin Coadministration vs Atorvastatin or Simvastatin Monotherapy in Patients at High Risk of CHD |  | 6 weeks |  |  |
| McKenney JMM | Comparative effects on lipid levels of combination therapy with a statin and extended-release niacin or ezetimibe versus a statin alone (the COMPELL study). | Atherosclerosis 2007 192 2 432 ballantin JM |  | 12 weeks |  |  |
| Meaney A | The vytorin on carotid intima-media thickness and overall arterial rigidity (VYCTOR) study | Journal of Clinical Pharmacology 2009 49:7 (838-847) |  |  | No outcome data |  |
| Melani L, Mills R, et al | Efficacy and safety of ezetimibe coadministered with pravastatin in patients with primary hypercholesterolemia: a prospective, randomized, double-blind trial. | Eur Heart J. 2003 Apr;24(8):717-28. |  | 12 weeks |  |  |
| Moutzouri E | Comparison of the effects of simvastatin vs. rosuvastatin vs. simvastatin⁄⁄ezetimibe on parameters of insulin resistance | Int J Clin Pract 2011 Moutzouri E Nov 65 11 1141-8 |  | 12 weeks |  |  |
| Musliner T | Efficacy and safety of ezetimibe 40 mg vs. ezetimibe 10 mg in the treatment of patients with homozygous sitosterolaemia | Efficacy and safety of ezetimibe 40 mg vs. ezetimibe 10 mg |  |  | No outcome data |  |
| Nakamura T | Co-administration of ezetimibe enhances proteinuria-lowering effects of pitavastatin in chronic kidney disease patients partly via a cholesterol-independent manner | Pharmacol Res 2010 61 58-61 Nakamura |  |  | No outcome data |  |
| Nakamura T et al | A comparison of the efficacy of combined ezetimibe and statin therapy with doubling of statin dose in patients with remnant lipoproteinemia on previous statin therapy | J Cardiol. 2012 Jul;60(1):12-7  [PMID: 22445441] |  |  |  | Type/dosage of comparator not evaluable |
| Nakou E | Statin-induced immunomodulation alters peripheral invariant natural killer T-cell prevalence in hyperlipidemic patients. | Cardiovascular drugs and therapy / sponsored by the International Society of Cardiovascular Pharmacotherapy, 2012, 26(4), 293 |  |  | No outcome data |  |
| Nakou ES | The effect of orlistat and ezetimibe, alone or in combination, on serum LDL and small dense LDL cholesterol levels in overweight and obese patients with hypercholesterolaemia | Curr Med Res Opin 2008 Nakou ES Jul 24 7 1919-29 |  |  | No outcome data |  |
| Nakou ES | The Effects of ezetimibe and/or orlistat on triglyceride-rich lipoprotein metabolism in obese hypercholesterolemic patients | Lipids 2010 45:5 (445-450) |  |  | No outcome data |  |
| NCT00129402 |  |  |  | 6 weeks |  |  |
| NCT00166504 |  |  |  | 6 weeks |  |  |
| NCT00202878 |  |  |  |  | No outcome data |  |
| NCT00276484 |  |  |  | 12 weeks |  |  |
| NCT00319449 |  |  |  | 6 weeks |  |  |
| NCT00413972 |  |  |  | 8 weeks |  |  |
| NCT00418834 |  |  |  | 12 weeks |  |  |
| NCT00423488 |  |  |  | 6 weeks |  |  |
| NCT00423579 |  |  |  | 6 weeks |  |  |
| NCT00442897 |  |  |  | 12 weeks |  |  |
| NCT00461630 |  |  |  |  |  | Not ezetimibe trial |
| NCT00462748 |  |  |  | 6 weeks |  |  |
| NCT00474123 |  |  |  | 6 weeks |  |  |
| NCT00477204 |  |  |  |  | No outcome data |  |
| NCT00481351 |  |  |  | 12 weeks |  |  |
| NCT00496730 |  |  |  | 8 weeks |  |  |
| NCT00525824 |  |  |  | 6 weeks |  |  |
| NCT00535405 |  |  |  | 12 weeks |  |  |
| NCT00548145 |  |  |  |  |  | Not ezetimibe trial |
| NCT00559962 |  |  |  | 12 weeks |  |  |
| NCT00639158 |  |  |  | 12 weeks |  |  |
| NCT00652327 |  |  |  | 8 weeks |  |  |
| NCT00653523 |  |  | not RCT |  |  |  |
| NCT00654095 |  |  | not RCT |  |  |  |
| NCT00654628 |  |  | not RCT |  |  |  |
| NCT00701727 |  |  |  | 7 weeks |  |  |
| NCT00738972 |  |  |  |  | No outcome data |  |
| NCT00762164 |  |  |  | 6 weeks |  |  |
| NCT00782184 |  |  |  | 6 weeks |  |  |
| NCT00783263 |  |  |  | 6 weeks |  |  |
| NCT00794677 |  |  |  | 6 weeks |  |  |
| NCT00810303 |  |  | not RCT |  |  |  |
| NCT00862251 |  |  |  | 6 weeks |  |  |
| NCT00867165 |  |  |  | 12 weeks |  |  |
| NCT00871351 |  |  |  | 16weeks |  |  |
| NCT01070953 |  |  |  | 4 weeks |  |  |
| NCT01070966 |  |  | not RCT |  |  |  |
| NCT01077830 |  |  | not RCT |  |  |  |
| NCT01154036 |  |  |  | 6 weeks |  |  |
| NCT01236430 |  |  |  |  |  | Bioequivalence study |
| NCT01274559 |  |  |  | 12 weeks |  |  |
| NCT01333436 |  |  |  |  |  | Physiopathology study |
| NCT01370590 |  |  |  | 6 weeks |  |  |
| NCT01370603 |  |  |  | 2 weeks |  |  |
| NCT01381679 |  |  | not RCT |  |  |  |
| NCT01414192 |  |  | not RCT |  |  |  |
| NCT01436253 |  |  | not RCT |  |  |  |
| NCT01611883 |  |  |  |  |  | Physiopathology study |
| Okada K | Long-term effects of ezetimibe-plus-statin therapy on low-density lipoprotein cholesterol levels as compared with double-dose statin therapy in patients with coronary artery disease | Atherosclerosis 2012 224 2 454 Okada K |  |  | No outcome data |  |
| Okada K | Clinical Usefulness of Additional Treatment With Ezetimibe in Patients With Coronary Artery Disease on Statin Therapy | Circ J 2011 75 2496-2504 Okada |  | 12 weeks |  |  |
| Olijhoek JK | The effects of low-dose simvastatin and ezetimibe compared to high-dose simvastatin alone on post-fat load endothelial function in patients with metabolic syndrome: a randomized double-blind crossover trial | Journal of cardiovascular pharmacology 2008 52 2 145 Olijhoek JK |  | 6 weeks |  |  |
| Ose L | A multi centre, randomised, double-blind 14-week extension study examining the long term safety and efficacy profile of ezetimibe/simvastatin combination tablet | Int J Clin Pract 2007 6191469-1480 |  | 14 weeks |  |  |
| Ose L | Effects of ezetimibe/simvastatin on lipoprotein subfractions in patients with primary hypercholesterolemia: an exploratory analysis of archived samples using two commercially available techniques. | Clinical therapeutics 2007 29 11 2419 Ose L |  | 12 weeks |  |  |
| Ostad MA | Flow-mediated dilation in patients with coronary artery disease is enhanced by high dose atorvastatin compared to combined low dose atorvastatin and ezetimibe: results of the CEZAR study | Atherosclerosis 2009 205 1 227 Ostad MA |  | 8 weeks |  |  |
| Patel JV | Efficacy, safety and LDL-C goal attainment of ezetimibe 10 mg-simvastatin 20 mg vs. placebo-simvastatin 20 mg in UK-based adults with coronary heart disease and hypercholesterolaemia | International journal of clinical practice 2006 60 8 914 Patel JV |  | 6 weeks |  |  |
| Pearson T | Effectiveness of the addition of ezetimibe to ongoing statin therapy in modifying lipid profiles and attaining low-density lipoprotein cholesterol goals in older and elderly patients: Subanalyses of data from a randomized, double-blind, placebo-controlled tria | The American journal of geriatric pharmacotherapy 2005 3 4 218 Pearson T |  | 6 weeks |  |  |
| Pearson TA | A community-based, randomized trial of ezetimibe added to statin therapy to attain NCEP ATP III goals for LDL cholesterol in hypercholesterolemic patients: the ezetimibe add-on to statin for effectiveness (EASE) trial | Mayo Clinic proceedings. Mayo Clinic 2005 80 5 587 Pearson TA |  | 6 weeks |  |  |
| Pearson TA | Effectiveness of ezetimibe added to ongoing statin therapy in modifying lipid profiles and low-density lipoprotein cholesterol goal attainment in patients of different races and ethnicities: a substudy of the Ezetimibe add-on to statin for effectiveness trial | Mayo Clinic proceedings. Mayo Clinic 2006 81 9 1177 Pearson TA |  | 6 weeks |  |  |
| Pesaro AEP | Pleiotropic effects of ezetimibe/simvastatin vs. high dose simvastatin | International journal of cardiology 2012 158 3 400 Pesaro AE |  | 6 weeks |  |  |
| Piorkowski M | Treatment With Ezetimibe Plus Low-Dose Atorvastatin Compared With Higher-Dose Atorvastatin Alone | J Am Coll Cardiol 2007 Piorkowski M Mar 13 49 10 1035-42 |  | 4weeks |  |  |
| Pisciotta L | Nutraceutical pill containing berberine versus ezetimibe on plasma lipid pattern in hypercholesterolemic subjects and its additive effect in patients with familial hypercholesterolemia on stable cholesterol-lowering treatment | Lipids in Health and Disease 2012 11 123 Pisciotta |  |  | No outcome data |  |
| Polis AB | Low-Density Lipoprotein Cholesterol Reduction and Goal Achievement With Ezetimibe/Simvastatin Versus Atorvastatin or Rosuvastatin in Patients With Diabetes, Metabolic Syndrome, or Neither Disease, Stratified by National Cholesterol Education Program Risk Category | Metabolic syndrome and related disorders 2009 7 6 601 Polis AB |  | 6 weeks |  |  |
| Prescott L.M | American Heart Association Scientific Session 2005 | P and T 2006 31:1 (48-51) |  |  | No outcome data |  |
| Quarta CC, et al | Safety and efficacy of ezetimibe with low doses of simvastatin in heart transplant recipients. | Heart Lung Transplant. 2008 Jun;27(6):685-8. doi: 10.1016/j.healun.2008.02.014. Epub 2008 Apr 28. | not RCT |  |  |  |
| Reyderman L | The effect of fluvastatin on the pharmacokinetics and pharmacodynamics of ezetimibe | Curr Med Res Opin 2005 8 1171-1179 |  | 2 weeks |  |  |
| Reyderman L et al | Pharmacokinetics of ezetimibe in subjects with normal renal function or severe chronic renal insufficiency | Clin Pharmacol Thet 71:MPI89A, 2002 (abs) | not RCT |  |  |  |
| Robinson J.G., | Efficacy and safety of ezetimibe and ezetimibe plus statin therapy in patients aged under 65, 65-74 and 75 years and older | Aging Health 2007 3:6 (691-705) | Not RCT |  |  |  |
| Robinson JG | Achievement of specified low-density lipoprotein cholesterol, non-high-density lipoprotein cholesterol apolipoprotein B, and high-sensitivity C-reactive protein levels with ezetimibe/simvastatin or atorvastatin in metabolic syndrome patients with and without atherosclerotic vascular disease (from the VYMET study) | Journal of clinical lipidology 2011 5 6 474 Robinson JG |  | 6 weeks |  |  |
| Robinson JG, et al | Lipid-altering efficacy and safety of ezetimibe/simvastatin versus atorvastatin in patients with hypercholesterolemia and the metabolic syndrome (from the VYMET study). | Am J Cardiol. 2009 Jun 15;103(12):1694-702. doi: 10.1016/j.amjcard.2009.05.003. |  | 6 weeks |  |  |
| Rodney RA, et al | Efficacy and safety of coadministration of ezetimibe and simvastatin in African-American patients with primary hypercholesterolemia. | J Natl Med Assoc 2006, 98:772-778. |  | 12 weeks |  |  |
| Roeters HWO | The efficacy of statin monotherapy uptitration versus switching to ezetimibe/ simvastatin: results of theEASEGO study* | Current Medical Research and Opinion® Vol. 24, No. 3, 2008, 685–694 |  | 12 weeks |  |  |
| Rosen JB, et al | Consistency of effect of ezetimibe/simvastatin compared with intensified lipidlowering treatment strategies in obese and non-obese diabetic subjects | Lipids Health Dis. 2013;12:103. [PMID: 23866306] |  | 12 weeks |  |  |
| Rosen JB, R, et al | A comparison of efficacy and safety of an ezetimibe/simvastatin combination compared with other intensified lipid-lowering treatment strategies in diabetic patients with symptomatic cardiovascular disease. | Diab Vasc Dis Res. 2013;10: 277-86. [PMID: 23288881] |  | 12 weeks |  |  |
| Rosolová H. | ENHANCE trial. Effect of combination ezetimibe and highdose simvastatin vs simvastatin alone on the atherosclerosis process in patients with heterozygous familial hypercholesterolemia | Cor et Vasa 2008 50:7-8 (306-308) |  |  |  | ENHANCE 2008 data |
| Rossebø AB et al | Design and baseline characteristics of the simvastatin and ezetimibe in aortic stenonis (SEAS) study | The American journal of cardiology 2007 99 7 970 Rossebø AB |  |  |  | SEAS 2008 data |
| Rotella CM | Ezetimibe/simvastatin vs simvastatin in coronary heart disease patients with or without diabetes | Rotella et al. Lipids in Health and Disease 2010, 9:80 |  | 6 weeks |  |  |
| Rudofsky G | Identical LDL-cholesterol lowering but non-identical effects on NF-κB activity: High dose simvastatin vs combination therapy with ezetimibe | Atherosclerosis 2012 223 1 190 Rudofsky G |  |  |  |  |
| Ruggenenti, P | Effects of Combined Ezetimibe and Simvastatin Therapy as Compared With Simvastatin Alone in Patients With Type 2 Diabetes | Diabetes Care 2010 33 1954–1956 Ruggenenti |  | 2 mesi |  |  |
| Russell, M | achieving lipid targets in adults with type 2 diabetes--the sands study | J Clin Lipidol. 2010 ; 4(5): 435–443 |  |  |  | Not ezetimibe trial |
| Sager PT | Effects of ezetimine coadministered with simvastatin on C-reactive protein in a large cohort of hypercholesterolomic patients | Atherosclerosis 2005 179 2 361 Sager PT |  | 12 weeks |  |  |
| Sager PT | Effect of coadmistration of ezetimibe and simvastatin on high sensitivity C-reactive protein | The American journal of cardiology 2003 92 12 1414 Sager PT |  | 12 weeks |  |  |
| Samaha FF | Inhibition of microsomal triglyceride transfer protein alone or with ezetimibe in patients with moderate hypercholesterolemia | Nat Clin Pract Cardiovasc Med 2008 Samaha FF Aug 5 8 497-505 |  | 12 weeks |  |  |
| Sasaki J | Double-dose pravastatin versus add-on ezetimibe with low-dose pravastatin - effects on LDL cholesterol, cholesterol absorption, and cholesterol synthesis in Japanese patients with hypercholesterolemia (PEAS study) | J Atheroscl Thromb 2012 19 485 Sasaki |  | 12 weeks |  |  |
| Settergren M, Bohm F, Ryden L, Pernow J. | Cholesterol lowering is more important than pleiotrophic effects of statins for endothelial function in patient with dysglycaemia and coronary artery disease. | Eur Heart J. 2008;29:1753-60. |  | 6 weeks |  |  |
| Shankar PK, et a | Efficacy and tolerability of fixed-dose combination of simvastatin plus ezetimibe in patients with primary hypercholesterolemia: Results of a multicentric trial from India | J Clin Lipidol 2007;1:264-70. |  | 12weeks |  |  |
| Shaw SM, et al | The efficacy and tolerability of ezetimibe in cardiac transplant recipients taking cyclosporin | Transplantation. 2009 Mar 15;87(5):771-5 [PMID: 19295325] |  |  |  | Type/dosage of comparator not evaluable |
| Simons L | Effects of ezetimibe added to on-going statin therapy on the lipid profile of hypercholesterolemic patients with diabetes mellitus or metabolic syndrome | Curr Med Res Opin 2004 20 1437-45 Simons |  | 8 weeks |  |  |
| Steg PG | A randomised trial of three counselling strategies for lifestyle changes in patients with hypercholesterolemia treated with ezetimibe on top of statin therapy (TWICE) | Archives of Cardiovascular Disease (2008) 101, 723—735 |  | 10 weeks |  |  |
| Stein EA | Effect of a monoclonal antibody to PCSK9, REGN727/SAR236553, to reduce low-density lipoprotein cholesterol in patients with heterozygous familial hypercholesterolaemia on stable statin dose with or without ezetimibe therapy: a phase 2 randomised controlled trial | Lancet 2012 380 9836 29 Stein EA |  | 20 weeks |  |  |
| Stein E | Achieving lipoprotein goals in patients at high risk with severe hypercholesterolemia : Efficacy and safety of ezetimibe co-administered with atorvastatin | American heart journal 2004 148 3 447 Stein E |  | 14 weeks |  |  |
| Strony J | Tolerability And Effects On Lipids Of Ezetimibe Coadministered With Pravastatin Or Simvastatin For Twelwe Months: Results From Two Open Laber Extension Studies In Hypercholesterolemic Patients | Clinical Therapeutics 30(12):2280 2008 | not RCT |  |  |  |
| Strony J, et al | Long-term safety and tolerability of ezetimibe coadministered with simvastatin in hypercholesterolemic patients: a randomized, 12-month double-blind extension study | Curr Med Res Opin. 2008 Nov;24(11):3149-57 [PMID: 18842166 ] |  |  |  | Type/dosage of comparator not evaluable |
| Suzuki H. | Comparative efficacy and adverse effects of the addition of ezetimibe to statin versus statin titration in chronic kidney disease patients | Therapeutic Advances in Cardiovascular Disease 2013 7:6 (306- 315) Therapeutic Advances in Cardiovascular Disease 2013 7:6 (306- 315) |  |  | No outcome data |  |
| Taylor AJ | Paradoxical progression of atherosclerosis related to low-density lipoprotein reduction and exposure to ezetimibe | Eur Heart J-2012-Taylor-2939-45 |  |  | No outcome data |  |
| Taylor AJ et al | ARBITER Extended-release niacin or ezetimibe and carotid intima-media thickness | N Engl J Med. 2009 Nov 26;361(22):2113-22 [PMID: 19915217] |  |  |  | inadequate comparison (not possible to assess the net effect of E) |
| Tendolkar I | One-year cholesterol lowering treatment reduces medial temporal lobe atrophy and memory decline in stroke-free elderly with atrial fibrillation: Evidence from a parallel group randomized trial | International Journal of Geriatric Psychiatry 2012 27:1 (49-58) |  |  | No outcome data |  |
| Teramoto T | Clinical Efficacy and Tolerability of Ezetimibe in Combination With Atorvastatin in Japanese Patients With Hypercholesterolemia—Ezetimibe Phase IV Randomized Controlled Trial in Patients With Hypercholesterolemia | Curr Ther Res Clin Exp. 2012;73:16–40 |  | 12 weeks |  |  |
| The AIM-HIGH Investigators (DUPLICATO N. 128) | Niacin in Patients with Low HDL Cholesterol Levels Receiving Intensive Statin Therapy | N Engl J Med 2011;365:2255-67. |  |  |  | Not ezetimibe trial |
| Thongtang N | Effects of ezetimibe added to statin therapy on markers of cholesterol absorption and synthesis and LDL-C lowering in hyperlipidemic patients | Atherosclerosis 2012 225 2 388 Thongtang N |  | 6 weeks |  |  |
| Tomassini JE | Effect of ezetimibe/simvastatin compared with atorvastatin on lipoprotein subclasses in patients with type 2 diabetes and hypercholesterolaemia | Diabetes, obesity & metabolism 2009 11 9 855 Tomassini JE |  | 6 weeks |  |  |
| Torimoto K | Efficacy of combination of Ezetimibe 10 mg and rosuvastatin 2.5 mg versus rosuvastatin 5 mg monotherapy for hypercholesterolemia in patients with type 2 diabetes | Torimoto et al. Lipids in Health and Disease 2013, 12:137 |  | 12 weeks |  |  |
| Tremblay AJ | Effects of ezetimibe and simvastatin on apolipoprotein B metabolism in males with mixed hyperlipidemia | J Lipid Res 2009 50 1463-71 Tremblay |  | 6 weeks |  |  |
| Tribble D | Effects of fenofibrate and ezetimibe, both as monotherapy and in coadministration, on cholesterol mass within lipoprotein subfractions and low-density lipoprotein peak particle size in patients with mixed hyperlipidemia | Metabolism clinical and experimental 2008 57 6 796 Tribble DL |  | 12 weeks |  |  |
| Uemura Y | Atorvastatin 10 mg plus ezetimibe 10 mg compared with atorvastatin 20 mg: Impact on the lipid profile in Japanese patients with abnormal glucose tolerance and coronary artery disease | J Cardiol 2012 Uemura Y Jan 59 1 50-6 |  |  | No outcome data |  |
| van Lennep HWO | The efficacy of statin monotherapy uptitration versus switching to ezetimibe/simvastatin: results of the EASEGO study | CMRO 2008 Roeters van Lennep HWO 24 3 685-94 |  | 15 weeks |  |  |
| Villines tc | The ARBITER 6-HALTS Trial (Arterial Biology for the Investigation of the Treatment Effects of Reducing Cholesterol 6– HDL and LDL Treatment Strategies in Atherosclerosis) Final Results and the Impact of Medication Adherence, Dose, and Treatment Duration | J Am Coll Cardiol 2010;55:2721–6) 2010 |  |  | No outcome data |  |
| Weinstock RS, | Effect of ezetimibe/simvastatin vs atorvastatin on lowering levels of LDL-C and non-HDL-C, Apo B, and hs-CRP in patients with type 2 diabetes. | J Clin Lipidology 2008;2:25-35. |  | 6 weeks |  |  |
| Westerweel PE, | Endothelial progenitor cell evels in obese men with metabolic syndrome and the effect of simvastatin monotherapy vs simvastatin/ezetimibe combination therapy. | Eur Heart J. 2008;29:2808-17. |  | 6 weeks |  |  |
| Winkler K | Fluvastatin/fenofibrate vs. simvastatin/ezetimibe in patients with metabolic syndrome: different effects on LDL-profiles | European journal of clinical investigation 2009 39 6 463 Winkler K |  | 12 weeks |  |  |
| Wohl DA | Ezetimibe Alone Reduces Low-Density Lipoprotein Cholesterol in HIV-Infected Patients Receiving Combination Antiretroviral Therapy | Clin Infect Dis.-2008-Wohl-1105-8 |  | 14 weeks |  |  |
| Yamazaki D | Comparison of anti-inflammatory effects and high-density lipoprotein cholesterol levels between therapy with quadruple-dose rosuvastatin and rosuvastatin combined with ezetimibe | Lipids in Health and Disease 2013 12 9 Yamazaki |  | 12 weeks |  |  |
| Yoon HS | Comparison of effects of morning versus evening administration of ezetimibe/simvastatin on serum cholesterol in patients with primary hypercholesterolemia | The Annals of pharmacotherapy 2011 45 7-8 841 Yoon HS |  | 12 weeks |  |  |
| Zema M.J. | Colesevelam HCl and ezetimibe combination therapy provides effective lipid-lowering in difficult-to-treat patients with hypercholesterolemia | American Journal of Therapeutics 2005 12:4 (306-310) |  | 18 weeks |  |  |
| Zieve F | Safety and Efficacy of Ezetimibe Added to Atorvastatin Versus Up Titration of Atorvastatin to 40 mg in Patients ≥65 Years of Age (from the ZETia in the ELDerly [ZETELD] Study) | The American journal of cardiology 2010 105 5 656 Zieve F |  | 12 weeks |  |  |
| Zieve F | Observed and predicted reduction of ischemic cardiovascular events in the Simvastatin and Ezetimibe in Aortic Stenosis trial | The American journal of cardiology 2010 105 12 1802 Holme I |  |  |  | SEAS 2008 data |
| Zinellu A | LDL S-homocysteinylation decrease in chronic kidney disease patients undergone lipid lowering therapy. | European journal of pharmaceutical sciences : official journal of the European Federation for Pharmaceutical Sciences, 2012, 47(1), 117 |  |  | No outcome data |  |
| Zubaid M | Effect of ezetimibe coadministration with simvastatin in a Middle Eastern population: a prospective, multicentre, randomized, double-blind, placebo-controlled trial | J Cardiovasc Med 2008 Zubaid M Jul 9 7 688-93 |  | 6 weeks |  |  |
