# Supplementary material for: Clinical Efficacy and Safety of Ezetimibe on Major Cardiovascular Endpoints: Systematic Review and Meta-Analysis of Randomized Controlled Trials
Source: PLoS One. 2015 Apr 27;10(4):e0124587. doi: 10.1371/journal.pone.0124587 (PMC4411142; doi:10.1371/journal.pone.0124587)
Supplement: S7 Appendix — (DOCX) [file pone.0124587.s008.docx]

**S7 Appendix**

**a) Tables A-G : Individual trial results: Main analysis (E/No-E drug versus same drug)**

E, Ezetimibe

A, Atorvastatin

R, Rosuvastatin

S, Simvastatin

Phen, Phenofibrate

Individual dosages are expressed as mg/daily

nr, not reported

nc, not calculable

NB: we calculated the Risk Ratios considering the Absolute Risks of events constructed with the number of randomized patients as denominator (see Missing data analysis in Methods)

| Table A in S7 Appendix | Year |  | Cancer Results | | |
| --- | --- | --- | --- | --- | --- |
|  |  | Comparisons | Risk Ratio | IC 95% lower | IC 95% upper |
| Arimura | 2012 | E10+A10  vs. A10 | nr | nr | nr |
| Kouvelos | 2013 | E10+R10  vs. R10 | 0.36 | 0.01 | 8.75 |
| ENHANCE | 2008 | E10+S80  vs. S80 | nr | nr | nr |
| UK-HARP-II | 2006 | E10+S20  vs. S20 | 11.11 | 0.62 | 198.29 |
| Ballantyne | 2004 | E10+A10  vs. A10 | nr | nr | nr |
| West | 2011 | E10+S40  vs. S40 | nr | nr | nr |
| McKenney | 2006 | E10+PHEN 160  vs. PHEN 160 | nr | nr | nr |

| Table B in S7 Appendix | Year | Comparisons | All Cause Death Results | | |
| --- | --- | --- | --- | --- | --- |
|  |  |  | Risk Ratio | IC 95% lower | IC 95% upper |
| Arimura | 2012 | E10+A10  vs. A10 | 3.00 | 0.13 | 70.30 |
| Kouvelos | 2013 | E10+R10  vs. R10 | 0.08 | 0.00 | 1.46 |
| ENHANCE | 2008 | E10+S80  vs. S80 | 2.03 | 0.19 | 22.33 |
| UK-HARP-II | 2006 | E10+S20  vs. S20 | 7.07 | 0.37 | 135.12 |
| Ballantyne | 2004 | E10+A10  vs. A10 | - | - | - |
| West | 2011 | E10+S40  vs. S40 | 1.25 | 0.14 | 11.31 |
| McKenney | 2006 | E10+PHEN 160  vs. PHEN 160 | 2.09 | 0.09 | 50.96 |

| Table C in S7 Appendix | Year | Comparisons | CV Death Results | | |
| --- | --- | --- | --- | --- | --- |
|  |  |  | Risk Ratio | IC 95% lower | IC 95% upper |
| Arimura | 2012 | E10+A10  vs. A10 | 3.00 | 0.13 | 70.30 |
| Kouvelos | 2013 | E10+R10  vs. R10 | 0.10 | 0.01 | 1.76 |
| ENHANCE | 2008 | E10+S80  vs. S80 | 2.03 | 0.19 | 22.33 |
| UK-HARP-II | 2006 | E10+S20  vs. S20 | 5.05 | 0.25 | 103.88 |
| Ballantyne | 2004 | E10+A10  vs. A10 | nc | nc | nc |
| West | 2011 | E10+S40  vs. S40 | nr | nr | nr |
| McKenney | 2006 | E10+PHEN 160  vs. PHEN 160 | nr | nr | nr |

| Table D in S7 Appendix | Year | Comparisons | Not CV Death* Results | | |
| --- | --- | --- | --- | --- | --- |
|  |  |  | Risk Ratio | IC 95% lower | IC 95% upper |
| Arimura | 2012 | E10+A10  vs. A10 | nc | nc | nc |
| Kouvelos | 2013 | E10+R10  vs. R10 | 0.36 | 0.01 | 8.75 |
| ENHANCE | 2008 | E10+S80  vs. S80 | nc | nc | nc |
| UK-HARP-II | 2006 | E10+S20  vs. S20 | 3.03 | 0.12 | 73.50 |
| Ballantyne | 2004 | E10+A10  vs. A10 | nc | nc | nc |
| West | 2011 | E10+S40  vs. S40 | nr | nr | nr |
| McKenney | 2006 | E10+PHEN 160  vs. PHEN 160 | nr | nr | nr |

*We calculated the number of not-CV deaths by subtracting the number of CV deaths from number of all-cause deaths reported per single trial.

| Table E in in S7 Appendix | Year | Comparisons | Myocardial Infarction Results | | |
| --- | --- | --- | --- | --- | --- |
|  |  |  | Risk Ratio | IC 95% lower | IC 95% upper |
| Arimura | 2012 | E10+A10  vs. A10 | nc | nc | nc |
| Kouvelos | 2013 | E10+R10  vs. R10 | 0.36 | 0.01 | 8.75 |
| ENHANCE | 2008 | E10+S80  vs. S80 | 1.53 | 0.26 | 9.07 |
| UK-HARP-II | 2006 | E10+S20  vs. S20 | nc | nc | nc |
| Ballantyne | 2004 | E10+A10  vs. A10 | nr | nr | nr |
| West | 2011 | E10+S40  vs. S40 | 3.00 | 0.16 | 55.55 |
| McKenney | 2006 | E10+PHEN 160  vs. PHEN 160 | nr | nr | nr |

| Table F in S7 Appendix | Year | Comparisons | Stroke Results | | |
| --- | --- | --- | --- | --- | --- |
|  |  |  | Risk Ratio | IC 95% lower | IC 95% upper |
| Arimura | 2012 | E10+A10  vs. A10 | 0.33 | 0.01 | 7.81 |
| Kouvelos | 2013 | E10+R10  vs. R10 | 1.08 | 0.07 | 17.07 |
| ENHANCE | 2008 | E10+S80  vs. S80 | 1.02 | 0.06 | 16.19 |
| UK-HARP-II | 2006 | E10+S20  vs. S20 | 3.03 | 0.12 | 73.50 |
| Ballantyne | 2004 | E10+A10  vs. A10 | nr | nr | nr |
| West | 2011 | E10+S40  vs. S40 | 3.86 | 0.22 | 68.48 |
| McKenney | 2006 | E10+PHEN 160  vs. PHEN 160 | nr | nr | nr |

| Table G in S7 Appendix | Year | Comparisons | SAES Results | | |
| --- | --- | --- | --- | --- | --- |
|  |  |  | Risk Ratio | IC 95% lower | IC 95% upper |
| Arimura | 2012 | E10+A10  vs. A10 | nr | nr | nr |
| Kouvelos | 2013 | E10+R10  vs. R10 | nr | nr | nr |
| ENHANCE | 2008 | E10+S80  vs. S80 | nr | nr | nr |
| UK-HARP-II | 2006 | E10+S20  vs. S20 | 1.45 | 0.95 | 2.23 |
| Ballantyne | 2004 | E10+A10  vs. A10 | 0.76 | 0.30 | 1.96 |
| West | 2011 | E10+S40  vs. S40 | nr | nr | nr |
| McKenney | 2006 | E10+PHEN 160  vs. PHEN 160 | 1.14 | 0.60 | 2.01 |

**b) Tables H-P: Individual trial results: Complementary analysis (E/Simvastatin versus placebo)**

NB: we calculated the Risk Ratios considering the Absolute Risks of events constructed with the number of randomized patients as denominator (see Missing data analysis in Methods)

| Table H in S7 Appendix | Year | Comparisons | Cancer Results | | |
| --- | --- | --- | --- | --- | --- |
|  |  |  | Risk Ratio | IC 95% lower | IC 95% upper |
| SHARP | 2011 | E10+S20  vs. Placebo | 0.99 | 0.87 | 1.12 |
| SEAS | 2008 | E10+S40  vs. Placebo | 1.48 | 1.11 | 1.97 |

| Table I in S7 Appendix | Year | Comparisons | All cause death Results | | |
| --- | --- | --- | --- | --- | --- |
|  |  |  | Risk Ratio | IC 95% lower | IC 95% upper |
| SHARP | 2011 | E10+S20  vs. Placebo | 1.02 | 0.95 | 1.09 |
| SEAS | 2008 | E10+S40  vs. Placebo | 1.03 | 0.80 | 1.34 |

| Table L in S7 Appendix | Year | Comparisons | CV death Results | | |
| --- | --- | --- | --- | --- | --- |
|  |  |  | Risk Ratio | IC 95% lower | IC 95% upper |
| SHARP | 2011 | E10+S20  vs. Placebo | 0.92 | 0.81 | 1.06 |
| SEAS | 2008 | E10+S40  vs. Placebo | 0.83 | 0.57 | 1.20 |

| Table M in S7 Appendix | Year | Comparisons | Not CV death* Results | | |
| --- | --- | --- | --- | --- | --- |
|  |  |  | Risk Ratio | IC 95% lower | IC 95% upper |
| SHARP | 2011 | E10+S20  vs. Placebo | 1.07 | 0.97 | 1.17 |
| SEAS | 2008 | E10+S40  vs. Placebo | 1.30 | 0.89 | 1.90 |

* We calculated the number of not-CV deaths by subtracting the number of CV deaths from the number of all-cause deaths reported per single trial.

| Table N in S7 Appendix | Year | Comparisons | Myocardial Infarction Results | | |
| --- | --- | --- | --- | --- | --- |
|  |  |  | Risk Ratio | IC 95% lower | IC 95% upper |
| SHARP | 2011 | E10+S20  vs. Placebo | 0.84 | 0.67 | 1.05 |
| SEAS | 2008 | E10+S40  vs. Placebo | 0.64 | 0.35 | 1.18 |

| Table O in S7 Appendix | Year | Comparisons | Stroke Results | | |
| --- | --- | --- | --- | --- | --- |
|  |  |  | Risk Ratio | IC 95% lower | IC 95% upper |
| SHARP | 2011 | E10+S20  vs. Placebo | 0.83 | 0.68 | 1.01 |
| SEAS | 2008 | E10+S40  vs. Placebo | 1.12 | 0.69 | 1.83 |

| Table P in S7 Appendix | Year | Comparisons | SAEs Results | | |
| --- | --- | --- | --- | --- | --- |
|  |  |  | Risk Ratio | IC 95% lower | IC 95% upper |
| SHARP | 2011 | E10+S20  vs. Placebo | 1.01 | 0.95 | 1.07 |
| SEAS | 2008 | E10+S40  vs. Placebo | 0.99 | 0.91 | 1.09 |
